# Supplementary material for: Discovery and application of insertion-deletion (INDEL) polymorphisms for QTL mapping of early life-history traits in Atlantic salmon
Source: BMC Genomics. 2010 Mar 8;11:156. doi: 10.1186/1471-2164-11-156 (PMC2838853; doi:10.1186/1471-2164-11-156)
Supplement: Additional file 2 — Information on developed 76 locus single-run INDEL panel in Atlantic salmon. Information on fluorescence labeling, primer concentrations, PCR pooling and links to alignments, INDEL motifs and GENESCAN (Burge and Karlin 1997) predictions of genes/exons are available in html format. [file 1471-2164-11-156-S2.ZIP › Additionalfile2/table2456.html]

Contig Alignment

```
Base	A B C D E F G H I J K L M N O P Q R S T U V W X Y Z a b c d e f g h i j k l m n o p q r s t u v w x y z A B C D E F G H I J K L M N O P Q   
1	. . . . . . . . . . . . . . . . . . . . . G . . . . . . . . . . . . . . . . . . . . . . . . . . . . . . . . . . . . . . . . . . . . . . .    
2	. . . . . . . . . . . . . . . . . . . . . G . . . . . . . . . . . . . . . . . . . . . . . . . . . . . . . . . . . . . . . . . . . . . . .    
3	. . . . . . . . . . . . . . . . . . . . . G . . . . . . . . . . . . . . . . . . . . . . . . . . . . . . . . . . . . . . . . . . . . . . .    
4	. . . . . . . . . . . . . . . . . . . . . G . . . . . . . . . . . . . . . . . . . . . . . . . . . . . . . . . . . . . . . . . . . . . . .    
5	. . . . . . . . . . . . . . . . . . . . . G . . . . . . . . . . . . . . . . . . . . . . . . . . . . . . . . . . . . . . . . . . . . . . .    
6	. . . . . . . . . . . . . . . . . . . . . A . . . . . . . . . . . . . . . . . . . . . . . . . . . . . . . . . . . . . . . . . . . . . . .    
7	. . . . . . . . . . . . . . . . . . . . . T . . . . . . . . . . . . . . . . . . . . . . . . . . . . . . . . . . . . . . . . . . . . . . .    
8	. . . . . . . . . . . . . . . . . . . . . A . . . . . . . . . . . . . . . . . . . . . . . . . . . . . . . . . . . . . . . . . . . . . . .    
9	. . . . . . . . . . . . . . . . . . . . . T . . . . . . . . . . . . . . . . . . . . . . . . . . . . . . . . . . . . . . . . . . . . . . .    
10	. . . . . . . . . . . . . . . . . . . . . T . . . . . . . . . . . . . . . . . . . . . . . . . . . . . . . . . . . . . . . . . . . . . . .    
11	. . . . . . . . . . . . . . . . . . . . . T . . . . . . . . . . . . . . . . . . . . . . . . . . . . . . . . . . . . . . . . . . . . . . .    
12	. . . . . . . . . . . . . . . . . . . . . C . . . . . . . . . . . . . . . . . . . . . . . . . . . . . . . . . . . . . . . . . . . . . . .    
13	. . . . . . . . . . . . . . . . . . . . . G . . . . . . . . . . . . . . . . . . . . . . . . . . . . . . . . . . . . . . . . . . . . . . .    
14	. . . . . . . . . . . . . . . . . . . . . T . . . . . . . . . . . . . . . . . . . . . . . . . . . . . . . . . . . . . . . . . . . . . . .    
15	. . . . . . . . . . . . . . . . . . . . . C . . . . . . . . . . . . . . . . . . . . . . . . . . . . . . . . . . . . . . . . . . . . . . .    
16	. . . . . . . . . . . . . . . . . . . . . G G . . . . . . . . . . . . . . . . . . . . . . . . . . . . . . . . . . . . . . . . . . . . . .    
17	. . . . . . . . . . . . . . . . . . . . . A A . . . . . . . . . . . . . . . . . . . . . . . . . . . . . . . . . . . . . . . . . . . . . .    
18	. . . . . . . . . . . . . . . . . . . . . T T . . . . . . . . . . . . . . . . . . . . . . . . . . . . . . . . . . . . . . . . . . . . . .    
19	. . . . . . . . . . . . . . . . . . . . . T T . . . . . . . . . . . . . . . . . . . . . . . . . . . . . . . . . . . . . . . . . . . . . .    
20	. . . . . . . . . . . . . . . . . . . . . C C . . . . . . . . . . . . . . . . . . . . . . . . . . . . . . . . . . . . . . . . . . . . . .    
21	. . . . . . . . . . . . . . . . . . . . . T T . . . . . . . . . . . . . . . . . . . . . . . . . . . . . . . . . . . . . . . . . . . . . .    
22	. . . . . . . . . . . . . . . . . . . . . G G . . . . . . . . . . . . . . . . . . . . . . . . . . . . . . . . . . . . . . . . . . . . . .    
23	. . . . . . . . . . . . . . . . . . . . . G G . . . . . . . . . . . . . . . . . . . . . . . . . . . . . . . . . . . . . . . . . . . . . .    
24	. . . . . . . . . . . . . . . . A . . . . A A . . . . . . . . . . . . . . . . . . . . . . . . . . . . . . . . . . . . . . . . . . . . . .    
25	. . . . . . . . . . . . . . . . A . . . . A A . . . . . . . . . . . . . . . . . . . . . . . . . . . . . . . . . . . . . . . . . . . . . .    
26	. . . . . . . . . . . . . . . . A . . . . A A . . . . . . . . . . . . . . . . . . . . . . . . . . . . . . . . . . . . . . . . . . . . . .    
27	. . . . . . . . . . . . . . . . G . . . . G G . . . . . . . . . . . . . . . . . . . . . . . . . . . . . . . . . . . . . . . . . . . . . .    
28	. . . . . . . . . . . . . . . . G . . . . G G . . . . . . . . . . . . . . . . . . . . . . . . . . . . . . . . . . . . . . . . . . . . . .    
29	. . . . . . . . . . . . . . . . G . . . . G G . . . . . . . . . . . . . . . . . . . . . . . . . . . . . . . . . . . . . . . . . . . . . .    
30	. . . . . . . . . . . . . . . . G . . . . G G . . . . . . . . . . . . . . . . . . . . . . . . . . . . . . . . . . . . . . . . . . . . . .    
31	. . . . . . . . . . . . A . . . A . . . . A A . . . . . . . . . . . . . . . . . . . . . . . . . . . . . . . . . . . . . . . . . . . . . .    
32	. A . . . . . . . . . A A A A A A A . . . A A A . . . . . A . . . . . . . . . . . . . . . . . . . . . . . . . . . . . . . . . . . . . . .    
33	. G . . . . . . . . . G G G G G G G . . . G G G . . . . . G . . . . . . . . . . . . . . . . . . . . . . . . . . . . . . . . . . . . . . .    
34	. T . . . . . . . . . T T T T T T T . . . T T T . . . . . T . . . . . . . . . . . . . . . . . . . . . . . . . . . . . . . . . . . . . . .    
35	. G . . . . . . . . . G G G G G G G . . . G G G . . . . . G . . . . . . . . . . . . . . . . . . . . . . . . . . . . . . . . . . . . . . .    
36	. G . . . . . . . . . G G G G G G G . . . G G G . . . . . G . . . . . . . . . . . . . . . . . . . . . . . . . . . . . . . . . . . . . . .    
37	. C . . . C . . . . . C C C C C C C . . . C C C . . . . . C . . . . . . . . . . . . . . . . . . . . . . . . . . . . . . . . . . . . . . .    
38	. T . . . T . . . . . T T T T T T T . . . T T T . . . . . T . . . . . . . . . . . . . . . . . . . . . . . . . . . . . . . . . . . . . . .    
39	. A . . . A . . . . . A A A A A A A . . . A A A . . . . . A . . . . . . . . . . . . . . . . . . . . . . . . . . . . . . . . . . . . . . .    
40	. G . . . G . . . . . G G G G G G G . . . G G G . . . . . G . . . . . . . . . . . . . . . . . . . . . . . . . . . . . . . . . . . . . . .    
41	. C . . . C . . . . . C C C C C C C . . . C C C . . . . . C . . . . . . . . . . . . . . . . . . . . . . . . . . . . . . . . . . . . . . .    
42	. T . . . T . . . . . T T T T T T T . . . T T T . . . . . T . . . . . . . . . . . . . . . . . . . . . . . . . . . . . . . . . . . . . . .    
43	. G . . . G . . . . . G G G G G G G . . . G G G . . . . . G . . . . . . . . . . . . . . . . . . . . . . . . . . . . . . . . . . . . . . .    
44	. C . . . C . . . . . C C C C C C C . . . C C C . . . . . C . . . . . . . . . . . . . . . . . . . . . . . . . . . . . . . . . . . . . . .    
45	. T . . . T . . . . . T T T T T T T . . . T T T . . . . . T . . . . . . . . . . . . . . . . . . . . . . . . . . . . . . . . . . . . . . .    
46	. T . . . T . . . . . T T T T T T T . . . T T T . . . . . T . . . . . . . . . . . . . . . . . . . . . . . . . . . . . . . . . . . . . . .    
47	. T . . . T . . . . . T T T T T T T . . . T T T . . . . . T . . . . . . . . . . . . . . . . . . . . . . . . . . . . . . . . . . . . . . .    
48	. G . . . G . . . . . G G G G G G G . . . G G G . . . . . G . . . . . . . . . . . . . . . . . . . . . . . . . . . . . . . . . . . . . . .    
49	. G . . . G . . . . . G G G G G G G . . . G G G . . . . . G . . . . . . . . . . . . . . . . . . . . . . . . . . . . . . . . . . . . . . .    
50	. A . . . A . . . . . A A A A A A A . . . A A A . . . . . A . . . . . . . . . . . . . . . . . . . . . . . . . . . . . . . . . . . . . . .    
51	. A . . . A . . . . . A A A A A A A . . . A A A . . . . . A . . . . . . . . . . . . . . . . . . . . . . . . . . . . . . . . . . . . . . .    
52	. A . . . A . . . . . A A A A A A A . . . A A A . . . . . A . . . . . . . . . . . . . . . . . . . . . . . . . . . . . . . . . . . . A . .    
53	. G . . . G . . . . . G G G G G G G . . . G G G . . . . . G . . . . . . . . . . . . . . . . . . . . . . . . . . . . . . . . . . . . G . .    
54	. T . . . T . . . . . T T T T T T T . . . T T T . . . . . T . . . . . . . . . . . . . . . . . . . . . . . . . . . . . . . . . . . . T . .    
55	. T . . . T . . . . . T T T T T T T . . . T T T . . . . . T . . . . . . . . . . . . . . . . . . . . . . . . . . . . . . . . . . . . T . .    
56	. A . . . A . A . . . A A A A A A A . . . A A A . . . . . A . . . . . . . . . . . . . . . . . . . . . . . . . . . . . . . . . . . . A . .    
57	. T . . . T . T . . . T T T T T T T . . . T T T . . . . . T . . . . . . . . . . . . . . . . . . . . . . . . . . . . . . . . . . . . T . .    
58	. C . . . C . C . . . C C C C C C C . . . C C C . . . . . C . . . . . . . . . . . . . . . . . . . . . . . . . . . . . . . . . . . . C . .    
59	. G . . . G . G . G G G G G G G G G . . . G G G . . . . G G . . . . . . . . . . . . . . . . . . . . . . . . . . . . . . . . . . . . G . .    
60	. A . . . A . A . A A A A A A A A A . . . A A A . . . . A A . . . . . . . . . . . . . . . . . . . . . . . . . . . . . . . . . . . . A . .    
61	. A . . . A . A . A A A A A A A A A . . . A A A . . . . A A . . . . . . . . . . . . . . . . . . . . . . . . . . . . . . . . . . . . A . .    
62	. G . . . G . G . G G G G G G G G G . . . G G G . . . . G G . . . . . . . . . . . . . . . . . . . . . . . . . . . . . . . . . . . . G . .    
63	. C . . . C . C . C C C C C C C C C . . . C C C . . . . C C . . . . . . . . . . . . . . . . . . . . . . . . . . . . . . . . . . . . C . .    
64	. A . . . A . A . A A A A A A A A A . . . A A A . . . . A A . . . . . . . . . . . . . . . . . . . . . . . . . . . . . . . . . . . . A . .    
65	. G . . . G . G . G G G G G G G G G . . . G G G . . . . G G . . . . . . . . . . . . . . . . . . . . . . . . . . . . . . . . . . . . G . .    
66	. A . . . A . A . A A A A A A A A A . . . A A A . . . . A A . . . . . . . . . . . . . . . . . . . . . . . . . . . . . . . . . . . . A . .    
67	. A . . . A . A . A A A A A A A A A . . . A A A . . . . A A . . . . . . . . . . . . . . . . . . . . . . . . . . . . . . . . . . . . A . .    
68	. A . . . A . A . A A A A A A A A A . . . A A A . . . . A A . . . . . . . . . . . . . . . . . . . . . . . . . . . . . . . . . . . . A . .    
69	. G . . . G G G . G G G G G G G G G . . . G G G . . . . G G . . . . . . . . . . . . . . . . . . . . . . . . . . . . . . . . . . . . G . .    
70	. T . . . T T T . T T T T T T T T T . . . T T T . . . . T T . . . . . . . . . . . . . . . . . . . . . . . . . . . . . . . . . . . . T . .    
71	. T . . . T T T . T T T T T T T T T . . . T T T . . . . T T . . . . . . . . . . . . . . . . . . . . . . . . . . . . . . . . . . . . T . .    
72	. T . . . T T T . T T T T T T T T T . . . T T T . . . . T T . . . . . . . . . . . . . . . . . . . . . . . . . . . . . . . . . . . . T . .    
73	. T . . . T T T . T T T T T T T T T . . . T T T . . . . T T . . . . . . . . . . . . . . . . . . . . . . . . . . . . . . . . . . . . T . .    
74	. C C C . C C C . C C C C C C C C C . . . C C C . . . . C C . . . . . . . . . . . . . . . . . . . . . . . . . . . . . . . . . . . . C . .    
75	. T T T . T T T . T T T T T T T T T . . . T T T . . . . T T . . . . . . . . . . . . . . . . . . . . . . . . . . . . . . . . . . . . T . .    
76	. T T T . T T T . T T T T T T T T T . . . T T T . . . . T T . . . . . . . . . . . . . . . . . . . . . . . . . . . . . . . . . . . . T . .    
77	. T T T . T T T . T T T T T T T T T . . . T T T . . . . T T . . . . . . . . . . . . . . . . . . . . . . . . . . . . . . . . . . . . T . .    
78	. C C C . C C C . C C C C C C C C C . . . C C C . . . . C C . . . . . . . . . . . . . . . . . . . . . . . . . . . . . . . . . . . . C . .    
79	. T T T . T T T . T T T T T T T T T . . . T T T . . . . T T . . . . . . . . . . . . . . . . . . . . . . . . . . . . . . . . . . . . T . .    
80	. C C C . C C C . C C C C C C C C C . . . C C C . . . . C C . . . . . . . . . . . . . . . . . . . . . . . . . . . . . . . . . . . . C . .    
81	. T T T . T T T . T T T T T T T T T . . . T T T . . . . T T . . . . . . . . . . . . . . . . . . . . . . . . . . . . . . . . . . . . T . .    
82	. G G G . G G G . G G G G G G G G G . . . G G G . G G . G G . . . . . . . . . . . . . . . . . . . . . . . . . . . . . . . . . . . . G . .    
83	. A A A . A A A . A A A A A A A A A . . . A A A . A A . A A . . . . . . . . . . . . . . . . . . . . . . . . . . . . . . . . . . . . A . .    
84	. A A A . A A A . A A A A A A A A A . . . A A A . A A . A A . . . . . . . . . . . . . . . . . . . . . . . . . . . . . . . . . . . . A . .    
85	. G G G . G G G . G G G G G G G G G . . . G G G . G G . G G . . . . . . . . . . . . . . . . . . . . . . . . . . . . . . . . . . . . G . .    
86	. T T T . T T T . T T T T T T T T T . . . T T T . T T . T T . . . . . . . . . . . . . . . . . . . . . . . . . . . . . . . . . . . . T . .    
87	. T T T . T T T . T T T T T T T T T . . . T T T . T T . T T . . . . . . . . . . . . . . . . . . . . . . . . . . . . . . . . . . . . T . .    
88	. G G G . G G G . G G G G G G G G G . . . G G G . G G . G G . . . . . . . . . . . . . . . . . . . . . . . . . . . . . . . . . . . . G . .    
89	. G G G . G G G . G G G G G G G G G . . . G G G . G G . G G . . . . . . . . . . . . . . . . . . . . . . . . . . . . . . . . . . . . G . .    
90	. C C C . C C C . C C C C C C C C C . . . C C C . C C . C C . . . . . . . . . . . . . . . . . . . . . . . . . . . . . . . . . . . . C . .    
91	. T T T . T T T . T T T T T T T T T . . . T T T . T T . T T . . . . . . . . . . . . . . . . . . . . . . . . . . . . . . . . . . . . T . .    
92	. A A A . A A A . A A A A A A A A A . . . A A A . A A . A A . . . . . . . . . . . . . . . . . . . . . . . . . . . . . . . . . . . . A . .    
93	. G G G . G G G . G G G G G G G G G . . . G G G . G G . G G . . . . . . . . . . . . . . . . . . . . . . . . . . . . . . . . . . . . G . .    
94	. C C C . C C C . C C C C C C C C C . . . C C C . C C . C C . . . . . . . . . . . . . . . . . . . . . . . . . . . . . . . . . . . . C . .    
95	. T T T . T T T . T T T T T T T T T . . . T T T . T T . T T . . . . . . . . . . . . . . . . . . . . . . . . . . . . . . . . . . . . T . .    
96	. A A A . A A A . A A A A A A A A A . . . A A A . A A . A A . . . . . . . . . . . . . . . . . . . . . . . . . . . . . . . . . . . . A . .    
97	. A A A . A A A . A A A A A A A A A . . . A A A . A A . A A . . . . . . . . . . . . . . . . . . . . . . . . . . . . . . . . . . . . A . .    
98	. T T T . T T T . T T T T T T T T T . . . T T T . T T . T T . . . . . . . . . . . . . . . . . . . . . . . . . . . . . . . . . . . . T . .    
99	. A A A . A A A . A A A A A A A A A . . . A A A . A A . A A . . . . . . . . . . . . . . . . . . . . . . . . . . . . . . . . . . . . A . .    
100	. A A A . A A A . A A A A A A A A A . . . A A A . A A . A A . . . . . . . . . . . . . . . . . . . . . . . . . . . . . . . . . . . . A . .    
101	. C C C . C C C . C C C C C C C C C . . . C C C . C C . C C . . . . . . . . . . . . . . . . . . . . . . . . . . . . . . . . . . . . C . .    
102	. A A A . A A A . A A A A A A A A A . . . A A A . A A . A A . . . . . . . . . . . . . . . . . . . . . . . . . . . . . . . . . . . . A . .    
103	. A A A . A A A . A A A A A A A A A . . . A A A . A A . A A . . . . . . . . . . . . . . . . . . . . . . . . . . . . . . . . . . . . A . .    
104	. A A A . A A A . A A A A A A A A A . . . A A A . A A . A A . . . . . . . . . . . . . . . . . . . . . . . . . . . . . . . . . . . . A . .    
105	. C C C . C C C . C C C C C C C C C . . . C C C . C C . C C . . . . . . . . . . . . . . . . . . . . . . . . . . . . . . . . . . . . C . .    
106	A A A A . A A A . A A A A A A A A A . A . A A A A A A . A A . . . . . . . . . . . . . . . . . . . . . . . . . . . . . . . . . . . . A . .    
107	G G G G . G G G . G G G G G G G G G . G . G G G G G G . G G . . . . . . . . . . . . . . . . . . . . . . . . . . . . . . . . . . . . G . .    
108	A A A A . A A A . A A A A A A A A A . A . A A A A A A . A A . . . . . . . . . . . . . . . . . . . . . . . . . . . . . . . . . . . . A . .    
109	A A A A . A A A . A A A A A A A A A . A . A A A A A A . A A . . . . . . . . . . . . . . . . . . . . . . . . . . . . . . . . . . . . A A .    
110	G G G G . G G G . G G G G G G G G G . G . G G G G G G . G G . . . . . . . . . . . . . . . . . . . . . . . . . . . . . . . . . . . . G G .    
111	A A A A . A A A . A A A A A A A A A . A . A A A A A A . A A . . . . . . . . . . . . . . . . . . . . . . . . . . . . . . . . . . . . A A .    
112	A A A A . A A A A A A A A A A A A A . A . A A A A A A . A A . . . . . . . . . . . . . . . . . . . . . . . . . . . . . . . . . . . . A A .    
113	A A A A . A A A A A A A A A A A A A . A . A A A A A A A A A . . . . . . . . . . . . . . . . . . . . . . . . . . . . . . . . . . . . A A .    
114	A A A A . A A A A A A A A A A A A A . A . A A A A A A A A A . . . . . . . . . . . . . . . . . . . . . . . . . . . . . . . . . . . . A A .    
115	A A A A . A A A A A A A A A A A A A . A . A A A A A A A A A . . . . . . . . . . . . . . . . . . . . . . . . . . . . . . . . . . . . A A .    
116	A A A A . A A A A A A A A A A A A A . A . A A A A A A A A A . . . . . . . . . . . . . . . . . . . . . . . . . . . . . . . . . . . . A A .    
117	A A A A . A A A A A A A A A A A A A . A . A A A A A A A A A . . . . . . . . . . . . . . . . . . . . . . . . . . . . . . . . . . . . A A .    
118	A A A A . A A A A A A A A A A A A A . A . A A A A A A A A A . . . . . . . . . . . . . . . . . . . . . . . . . . . . . . . . . . . . A A .    
119	- - - - . - - - - - - - - - - - - - . - . - A - - - - A - - . . . . . . . . . . . . . . . . . . . . . . . . . . . . . . . . . . . . - - .    
120	C C C C . C C C C C C C C C C C C C . C . C C C C C C C C C . . . . . . . . . . . . . . . . . . . . . . . . . . . . . . . . . . . . C C .    
121	A A A A . A A A A A A A A A A A A A . A . A A A A A A A A A . . . . . . . . . . . . . . . . . . . . . . . . . . . . . . . . . . . . A A .    
122	A A A A . A A A A A A A A A A A A A . A . A A A A A A A A A . . . . . . . . . . . . . . . . . . . . . . . . . . . . . . . . . . . . A A .    
123	C C C C . C C C C C C C C C C C C C . C . C C C C C C C C C . . . . . . . . . . . . . . . . . . . . . . . . . . . . . . . . . . . . C C .    
124	A A A A . A A A A A A A A A A A A A . A . A A A A A A A A A . . . . . . . . . . . . . . . . . . . . . . . . . . . . . . . . . . . . A A .    
125	- - - - . - - A - - - - A - - - A - . - . A - - - - - A A - . . . . . . . . . . . . . . . . . . . . . . . . . . . . . . . . . . . . A A .  8	 indel  
126	- - - - . - - C - - - - C - - - C - . - . C - - - - - C C - . . . . . . . . . . . . . . . . . . . . . . . . . . . . . . . . . . . . C C .  8	 indel  
127	- - - - . - - A - - - - A - - - A - . - . A - - - - - A A - . . . . . . . . . . . . . . . . . . . . . . . . . . . . . . . . . . . . A A .  8	 indel  
128	T T T T . T T T T T T T T T T T T T . T . T T T T T T T T T . . . . . . . . . . . . . . . . . . . . . . . . . . . . . . . . . . . . T T .    
129	G G G G . G G G G G G G G G G G G G . G . G G G G G G G G G . . . . . . . . . . . . . . . . . . . . . . . . . . . . . . . . . . . . G G .    
130	G G G G . G G G G G G G G G G G G G . G . G G G G G G G G G . . . . . . . . . . . . . . . . . . . . . . . . . . . . . . . . . . . . G G .    
131	G G G G . G G G G G G G G G G G G G . G . G G G G G G G G G . . . . . . . . . . . . . . . . . . . . . . . . . . . . . . . . . . . . G G .    
132	A G A A . A A A A A A A A A A A A A . A . A A A A A A A A A . . . . . . . . . . . . . . . . . . . . . . . . . . . . . . . . . . . . A A .    
133	A A A A . A A A A A A A A A A A A A . A . A A A A A A A A A . . . . . . . . . . . . . . . . . . . . . . . . . . . . . . . . . . A . A A .    
134	C C C C C C C C C C C C C C C C C C . C . C C C C C C C C C . . . . . . . . . . . . . . . . . . . . . . . . . . . . . . . . . . C . C C .    
135	A A A A A A A A A A A A A A A A A A . A . A A A A A A A A A . . . . . . . . . . . . . . . . . . . . . . . . . . . . . . . . . . A . A A .    
136	A A A A A A A A A A A A A A A A A A . A . A A A A A G A A A . . . . . . . . . . . . . . . . . . . . . . . . . . . . . . . . . . A . A A .    
137	C C C C C C C C C C C C C C C C C C . C C C C C C C C C C C . . . . . . . . . . . . . . . . . . . . . . . . . . . . . . . . . . C . C C .    
138	A A A A A A A A A A A A A A A A A A . A A A A A A A A A A A . . . . . . . . . . . . . . . . . . . . . . . . . . . . . . . . . . A . A A .    
139	G G G G G G G G G G G G G G G G G G . G G G G G G G G G G G . . . . . . . . . . . . . . . . . . . . . . . . . . . . . . . . . . G . G G .    
140	C C C C C C C C C C C C C C C C C C . C C C C C C C C C C C . . . . . . . . . . . . . . . . . . . . . . . . . . . . . . . . . . C . C C .    
141	A A A A A A A A A A A A A A A A A A . A A A A A A A A A A A . . . . . . . . . . . . . . . . . . . . . . . . . . . . . . . . . . A . A A .    
142	A A A A A A A A A A A A A A A A A A . A A A A A A A A A A A . . . . . . . . . . . . . . . . . . . . . . . . . . . . . . . . . . A . A A .    
143	G G G G G G G G G G G G G G G G G G . G G G G G G G G G G G . . . . . . . . . . . . . . . . . . . . . . . . . . . . . . . . . . G . G G .    
144	T T T T T T T T T T T T T T T T T T . T T T T T T T T T T T . . . . . . . . . . . . . . . . . . . . . . . . . . . . . . . . . . T . T T .    
145	C C C C C C C C C C C C C C C C C C . C C C C C C C C C C C . . . . . . . . . . . . . . . . . . . . . . . . . . . . . . . . . . C . C C .    
146	A A A A A A A A A A A A A A A A A A . A A A A A A A A A A A . . . . . . . . . . . . . . . . . . . . . . . . . . . . . . . . . . A . A A .    
147	A A A A A A A A A A A A A A A A A G . A A A A A A A A A A A . . . . . . . . . . . . . . . . . . . . . . . . . . . . . . . . . . A . A A .    
148	T T T T T T T T T T T T T T T T T T . T T T T T T T T T T T . . . . . . . . . . . . . . . . . . . . . . . . . . . . . . . . . . T . T T .    
149	T T T T T T T T T T T T T T T T T T . T T T T T T T T T T T . . . . . . . . . . . . . . . . . . . . . . . . . . . . . . . . . . T . T T .    
150	A A A A A A A A A A A A A A A A A A . A A A A A A A A A A A . . . . . . . . . . . . . . . . . . . . . . . . . . . . . . . . . . A . A A .    
151	A A A A A A A A A A A A A A A A A A . A A A A A A A A A A A . . . . . . . . . . . . . . . . . . . . . . . . . . . . . . . . . . A . A A .    
152	G G G G G G G G G G G G G G G G G G . G G G G G G G G G G G . . . . . . . . . . . . . . . . . . . . . . . . . . . . . . . . . . G . G G .    
153	G G G G G G G G G G G G G G G G G G . G G G G G G G G G G G . . . . . . . . . . . . . . . . . . . . . . . . . . . . . . . . . . G . G G .    
154	A A A A A A A A A A A A A A A A A A . A A A A A A A A A A A . . . . . . . . . . . . . . . . . . . . . . . . . . . . . . . . . . A . A A .    
155	A A A A A A A A A A A A A A A A A A . A A A A A A A A A A A . . . . . . . . . . . . . . . . . . . . . . . . . . . . . . . . . . A . A A .    
156	G G G G G G G G G G G G G G G G G G . G G G G G G G G G G G . . . . . . . . . . . . . . . . . . . . . . . . . . . . . . . . . . G . G G .    
157	G G G G G G G G G G G G G G G G G G . G G G G G G G G G G G . . . . . . . . . . . . . . . . . . . . . . . . . . . . . . . . . . G . G G .    
158	A A A A A A A A A A A A A A A A A A . A A A A A A A A A A A . . . . . . . . . . . . . . . . . . . . . . . . . . . . . . . . . . A . A A .    
159	T T T T T T T T T T T T T T T T T T . T T T T T T T T T T T . . . . . . . . . . . . . . . . . . . . . . . . . . . . . . . . . . T . T T .    
160	C C C C C C C C C C C C C C C C C C . C C C C C C C C C C C . . . . . . . . . . . . . . . . . . . . . . . . . . . . . . . . . . C . C C .    
161	T T T T T T T T T T T T T T T T T T . T T T T T T T T T T T . . . . . . . . . . . . . . . . . . . . . . . . . . . . . . . . . . T . T T .    
162	G G G G G G G G G G G G G G G G G G . G G G G G G G G G G G . . . . . . . . . . . . . . . . . . . . . . . . . . . . . . . . . . G . G G .    
163	C C C C C C C C C C C C C C C C C C . C C C C C C C C C C C . . . . . . . . . . . . . . . . . . . . . . . . . . . . . . . . . . C . C C .    
164	T T T T T T T T T T T T T T T T T T . T T T T T T T T T T T . . . . . . . . . . . . . . . . . . . . . . . . . . . . . . . . . . T . T T .    
165	C C C C C C C C C C C C C C C C C C . C C C C C C C C C C C . . . . . . . . . . . . . . . . . . . . . . . . . . . . . . . . . . C . C C .    
166	T T T T T T T T T T T T T T T T T T . T T T T T T T T T T T . . . . . . . . . . . . . . . . . . . . . . . . . . . . . . . . . . T . T T .    
167	C C C C C C C C C C C C C C C C C C . C C C C C C C C C C C . . . . . . . . . . . . . . . . . . . . . . . . . . . . . . . . . . C . C C .    
168	A A A A A A A A A A A A A A A A A A . A A A A A A A A A A A . . . . . . . . . . . . . . . . . . . . . . . . . . . . . . . . . . A . A A .    
169	G G G G G G G G G G G G G G G G G G . G G G G G G G G G G G . . . . . . . . . . . . . . . . . . . . . . . . . . . . . . . . . G G G G G .    
170	A A A A A A A A A A A A A A A A A A . A A A A A A A A G A A . . . . . . . . . . . . . . . . . . . . . . . . . . . . . . . . . A A A A A .    
171	A A A A A A A A A A A A A A A A A A . A A A A A A A A A A A . . . . . . . . . . . . . . . . . . . . . . . . . . . . . . . . . A A A A A .    
172	T T T T T T T T T T T T T T T T T T . T T T T T T T T T T T . . . . . . . . . . . . . . . . . . . . . . . . . . . . . . . . . T T T T T .    
173	A A A A A A A A A A A A A A A A A A . A A A A A A A A A A A . . . . . . . . . . . . . . . . . . . . . . . . . . . . . . . . . A A A A A .    
174	T T T T T T T T T T T T T T T T T T . T T T T T T T T T T T . . . . . . . . . . . . . . . . . . . . . . . . . . . . . . . . . T T T T T .    
175	C C C C C C C C C C C C C C C C C C . C C C C C C C C C C C . . . . . . . . . . . . . . . . . . . . . . . . . . . . . . . . . C C C C C .    
176	A A A A A A A A A A A A A A A A A A . A A A A A A A A A A A . . . . . . . . . . . . . . . . . . . . . . . . . . . . . . . . . A A A A A .    
177	G G G G G G G G G G G G G G G G G G . G G G G G G G G G G G . . . . . . . . . . . . . . . . . . . . . . . . . . . . . . . . . G G G G G .    
178	G G G G G G G G G G G G G G G G G G . G G G G G G G G G G G . . . . . . . . . . . . . . . . . . . . . . . . . . . . . . . . . G G G G G .    
179	A A A A A A A A A A A A A A A A A A . A A A A A A A A A A A . . . . . . . . . . . . . . . . . . . . . . . . . . . . . . . . . A A A A A .    
180	G G G G G G G G G G G G G G G G G G . G G G G G G G G G G G . . . . . . . . . . . . . . . . . . . . . . . . . . . . . . . . . G G G G G .    
181	C C C C C C C C C C C C C C C C C C . C C C C C C C C C C C . . . . . . . . . . . . . . . . . . . . . . . . . . . . . . . . . C C C C C .    
182	T T T T T T T T T T T T T T T T T T . T T T T T T T T T T T . . . . . . . . . . . . . . . . . . . . . . . . . . . . . . . . T T T T T T .    
183	G G G G G G G G G G G G G G G G G G . G G G G G G G G G G G . . . . . . . . . . . . . . . . . . . . . . . . . . . . . . . . G G G G G G .    
184	A A A A A A A A A A A A A A A A A A . A A A A A A A A A A A . . . . . . . . . . . . . . . . . . . . . . . . . . . . . . . . A A A A A A .    
185	C C C C C C C C C C C C C C C C C C . C C C C C C C C C C C . . . . . . . . . . . . . . . . . . . . . . . . . . . . . . . . C C C C C C .    
186	G G G G G G G G G G G G G G G G G G . G G G G G G G G G G G . . . . . . . . . . . . . . . . . . . . . . . . . . . . . . . . G G G G G G .    
187	T T T T T T T T T T T T T T T T T T . T T T T T T T T T T T . . . . . . . . . . . . . . . . . . . . . . . . . . . . . . . . T T T T T T .    
188	T T T T T T T T T T T T T T T T T T . T T T T T T T T T T T . . . . . . . . . . . . . . . . . . . . . . . . . . . . . . . . T T T T T T .    
189	T T T T T T T T T T T T T T T T T T . T T T T T T T T T T T . . . . . . . . . . . . . . . . . . . . . . . . . . . . . . . . T T T T T T .    
190	C C C C C C C C C C C C C C C C C C . C C C C C C C C C C C . . . . . . . . . . . . . . . . . . . . . . . . . . . . . . . . C C C C C C .    
191	T T T T T T T T T T T T T T T T T T . T T T T T T T T T T T . . . . . . . . . . . . . . . . . . . . . . . . . . . . . . . . T T T T T T .    
192	T T T T T T T T T T T T T T T T T T . T T T T T T T T T T T . . . . . . . . . . . . . . . . . . . . . . . . . . . . . . . . T T T T T T .    
193	A A A A A A A A A A A A A A A A A A . A A A A A A A A A A A . . . . . . . . . . . . . . . . . . . . . . . . . . . . . . . . A A A A A A .    
194	C C C C C C C C C C C C C C C C C C . C C C C C C C C C C C . . . . . . . . . . . . . . . . . . . . . . . . . . . . . . . . C C C C C C .    
195	A A A A A A A A A A A A A A A A A A . A A A A A A A A A A A . . . . . . . . . . . . . . . . . . . . . . . . . A . . . . . . A A A A A A .    
196	A A A A A A A A A A A A A A A A A A . A A A A A A A A A A A . . . . . . . . . . . . . . . . . . . . . . . . . A . . . . . . A A A A A A .    
197	A A A A A A A A A A A A A A A A A A . A A A A A A A A A A A . . . . . . . . . . . . . . . . . . . . . . . . . A . . . . . . A A A A A A .    
198	G G G G G G G G G G G G G G G G G G . G G G G G G G G G G G . . . . . . . . . . . . . . . . . . . . . . . . . G . . . . . . G G G G G G .    
199	C C C C C C C C C C C C C C C C C C . C C C C C C C C C C C . . . . . . . . . . . . . . . . . . . . . . . . . C . . . . . . C C C C C C .    
200	A A A A A A A A A A A A A A A A A A . A A A A A A A A A A A . . . . . . . . . . . . . . . . . . . . . . . . . A . . . . . . A A A A A A .    
201	A A A A A A A A A A A A A A A A A A . A A A A A A A A A A A . . . . . . . . . . . . . . . . . . . . . . . . . A . . . . . . A A A A A A .    
202	G G G G G G G G G G G G G G G G G G . G G G G G G G G G G G . . . . . . . . . . . . . . . . . . . . . . . . . G . . . . . . G G G G G G .    
203	A A A A A A A A A A A A A A A A A A . A A A A A A A A A A A . . . . . . . . . . . . . . . . . . . . . . . . . A . . . . . . A A A A A A .    
204	A A A A A A A A A A A A A A A A A A . A A A A A A A A A A A . . . . . . . . . . . . . . . . . . . . . . . . . A . . . . . . A A A A A A .    
205	A A A A A A A A A A A A A A A A A A . A A A A A A A A A A A . . . . . . . . . . . . . . . . . . . . . . . . . A . . . . . . A A A A A A .    
206	T T T T T T T T T T T T T T T T T T . T T T T T T T T T T T . . . . . . . . . . . . . . . . . . . . . . . . . T . . . . . . T T T T T T .    
207	T T T T T T T T T T T T T T T T T T . T T T T T T T T T T T . . . . . . . . . . . . . . . . . . . . . . . . . T . . . . . . T T T T T T .    
208	A A A A A A A A A A A A A A A A A A . A A A A A A A A A A A . . . . . . . . . . . . . . . . . . . . . . . . . A . . . . . . A A A A A A .    
209	T T T T T T T T T T T T T T T T T T . T T T T T T T T T T T . . . . . . . . . . . . . . . . . . . . . . . . . T . . . . . . T T T T T T .    
210	T T T T T T T T T T T T T T T T T T . T T T T T T T T T T T . . . . . . . . . . . . . . . . . . . . . . . . . T . . . . . . T T T T T T .    
211	C C C C C C C C C C C C C C C C C C . C C C C C C C C C C C . . . . . . . . . . . . . . . . . . . . . . . . . C . . . . . . C C C C C C .    
212	T T T T T T T T T T T T T T T T T T . T T T T T T T T T T T . . . . . . . . . . . . . . . . . . . . . . . . . T . . . . . . T T T T T T .    
213	G G G G G G G G G G G G G G G G G G . G G G G G G G G G G G . . . . . . . . . . . . . . . . . . . . . . . . . G . . . . . . G G G G G G .    
214	G G G G G G G G G G G G G G G G G G . G G G G G G G G G G G . . . . . . . . . . . . . . . . . . . . . . . . . G . . . . . . G G G G G G .    
215	C C C C C C C C C C C C C C C C C C . C C C C C C C C C C C . . . . . . . . . . . . . . . . . . . . . . . . . C . . . . . . C C C C C C .    
216	A A A A A A A A A A A A A A A A A A . A A A A A A A A A A A . . . . . . . . . . . . . . . . . . . . . . . . . A . . . . . . A A A A A A .    
217	C C C C C C C C C C C C C C C C C C . C C C C C C C C C C C . . . . . . . . . . . . . . . . . . . . . . . . . C . . . . . C C C C C C C .    
218	A A A A A A A A A A A A A A A A A A . A A A A A A A A A A A . . . . . . . . . . . . . . . . . . . . . . . . . A . . . . . A A A A A A A .    
219	C C C C C C C C C C C C C C C C C C . C C C C C C C C C C C . . . . . . . . . . . . . . . . . . . . . . . . . C . . . . . C C C C C C C .    
220	A A A A A A A A A A A A A A A A A A . A A A A A A A A A A A . . . . . . . . . . . . . . . . . . . . . . A . . A . . A . . A A A A A A A .    
221	A A A A A A A A A A A A A A A A A A . A A A A A A A A A A A . . . . . . . . . . . . . . . . . . . . . . A . . A . . A . . A A A A A A A .    
222	G G G G G G G G G G G G G G G G G G . G G A G G G G G G G G . . . . . . . . . . . . . . . . . . . . . . G . . G . . G . . G G A G G G G .    
223	A A A A A A A A A A A A A A A A A A . A A A A A A A A A A A . . . . . . . . . . . . . . . . . . . . . . A . . A . . A . . A A A A A A A A    
224	G G G G G G G G G G G G G G G G G G . G G G G G G G G G G G . . . . . . . . . . . . . . . . . . . . . . G . . G . . G . G G G G G G G G G    
225	A A A A A A A A A A A A A A A A A A . A A A A A A A A A A A . . . . . . . . . . . . . . . . . . . . . . A . . A . . A . A A A A A A A A A    
226	T T T T T T T T T T T T T T T T T T . T T T T T T T T T T T . . . . . . . . . . . . . . . . . . . . . . T . . T . . T . T T T T T T T T T    
227	T T T T T T T T T T T T T T T T T T . T T T T T T T T T T T . . . . . . . . . . . . . . . . . . . . . . T . . T . . T . T T T T T T T T T    
228	C C C C C C C C C C C C C C C C C C . C C C C C C C C C C C . . . . . . . . . . . . . . . . . . . . . . C . . C . . C . C C C C C C C C C    
229	A A A A A A A A A A A A A A A A A A . A A A A A A A A A A A . . . . . . . . . . . . . . . . . . . . . . A . . A . . A . A A A A A A A A A    
230	- - - - - - - - - - - - - - - - - - . - - - - - - - - - - - . . . . . . . . . . . . . . . . . . . . . . - . . - . . - . - - - A - - - - -    
231	G G G G G G G G G G G G G G G G G G . G G G G G G G G G G G . . . . . . . . . . . . . . . . . . . . . . G . . G . . G . G G G G G G G G G    
232	T T T T T T T T T T T T T T T T T T . T T T T T T T T T T T . . . . . . . . . . . . . . . . . . . . . . T . . T . . T . T T T T T T T T T    
233	G G G G G G G G G G G G G G G G G G . G G G G G G G G G G G . . . . . . . . . . . . . . . . . . . . . . G . . G . . G . G G G G G G G G G    
234	A A A A A A A A A A A A A A A A A A . A A A A A A A A A A A . . . . . . . . . . . . . . . . . . . . . . A . . A . . A . A A A A A A A A A    
235	A A A A A A A A A A A A A A A A A A . A A A A A A A A A A A . . . . . . . . . . . . . . . . . . . . . . A . . A . . A . A A A A A A A A A    
236	C C C C C C C C C C C C C C C C C C . C C C C C C C C C C C . . . . . . . . . . . . . . . . . . . . . . C . . C . . C . C C C C C C C C C    
237	T T T T T T T T T T T T T T T T T T . T T T T T T T T T T T . . . . . . . . . . . . . . . . . . . . . . T . . T . . T . T T T T T T T T T    
238	C C C C C C C C C C C C C C C C C C . C C C C C C C C C C C . . . . . . . . . . . . . . . . . . . . . . C . . C . . C . C C C C C C C C C    
239	C C C C C C C C C C C C C C C C C C . C C C C C C C C C C C . . . . . . . . . . . . . . . . . . . . . . C . . C . . C . C C C C C C C C C    
240	T T T T T T T T T T T T T T T T T T . T T T T T T T T T T T . . . . . . . . . . . . . . . . . . . . . . T . . T . . T . T T T T T T T T T    
241	C C C C C C C C C C C C C C C C C C . C C C C C C C C C C C . . . . . . . . . . . . . . . . . . . . . . C . . C . . C . C C C C C C C C C    
242	T T T T T T T T T T T T T T T T T T . T T T T T T T T T T T . . . . . . . . . . . . . . . . . . . . . . T . . T . . T . T T T T T T T T T    
243	C C C C C C C C C C C C C C C C C C . C C C C C C C C C C C . . . . . . . . . . . . . . . . . . . . . . C . . C . . C . C C C C C C C C C    
244	C C C C C C C C C C C C C C C C C C . C C C C C C C C C C C . . . . . . . . . . . . . . . . . . . . . . C . . C . . C . C C C C C C C C C    
245	A A A A A A A A A A A A A A A A A A A A A A A A A A A A A A . . . . . . . . . . . . . . . . . . . . . . A . . A . . A . A A A A A A A A A    
246	A A A A A A A A A A A A A A A A A A A A A A A A A A A A A A . . . . . . . . . . . . . . . . . . . . . . A . . A . . A . A A A A A A A A A    
247	A A A A A A A A A A A A A A A A A A A A A A A A A A A A A A . . . . . . . . . . . . . . . . . . . . . . A . . A . . A . A A A A A A A A A    
248	G G G G G G G G G G G G G G G G G G G G G G G G G G G G G G . . . . . . . . . . . . . . . . . . . . . . G . G G . . G . G G G G G G G G G    
249	A A A A A A A A A A A A A A A A A A A A A A A A A A A A A A . . . . . . . . . . . . . . . . . . . . . . A . A A . . A . A A A A A A A A A    
250	G G G G G G G G G G G G G G G G G G G G G G G G G G G G G G . . . . . . . . . . . . . . . . . . . . . . G . G G . . G . G G G G G G G G G    
251	G G G G G G G G G G G G G G G G G G G G G G G G G G G G G G . . . . . . . . . . . . . . . . . . . . . . G . G G . . G . G G G G G G G G G    
252	A A A A A A A A A A A A A A A A A A A A A A A A A A A A A A . . . . . . . . . . . . . . . . . . . . . . A . A A . . A . A A A A A A A A A    
253	C C C C C C C C C C C C C C C C C C C C C C C C C C C C C C . . . . . . . . . . . . . . . . . . . . . . C . C C . . C . C C C C C C C C C    
254	A A A A A A A A A A A A A A A A A A A A A A A A A A G A A A . . . . . . . . . . . . . . . . . . . . . . A . A A . . A . A A A A A A A A A    
255	G G G G G G G G G G G G G G G G G G G G G G G G G G G G G G . . . . . . . . . . . . . . . . . . . . . . G . G G G . G . G G G G G G G G G    
256	A A A A A A A A A A A A A A A A A A A A A A A A A A A A A A . . . . . . . . . . . . . . . . . . . . . . A . A A A . A . A A A A A A A A A    
257	A A A A A A A A A A A A A A A A A A A A A A A A A A A A A A . . . . . . . . . . . . . . . . . . . . . . A . A A A A A . A A A A A A A A A    
258	A A A A A A A A A A A A A A A A A A A A A A A A A A A A A A . . . . . . . . . . . . . . . . . . . . . . A . A A A A A . A A A A A A A A A    
259	C C C C C C C C C C C C C C C C C C C C C C C C C C C C C C . . . . . . . . . . . . . . . . . . . . . . C . C C C C C . C C C C C C C C C    
260	A A A A A A A A A A A A A A A A A A A A A A A A A A A A A A . . . . . . . . . . . . . . . . . . . . . . A . A A A A A . A A A A A A A A A    
261	T T T T T T T T T T T T T T T T T T T T T T T T T T T T T T . . . . . . . . . . . . . . . . . . . . . . T . T T T T T . T T T T T T T T T    
262	C C C C C C C C C C C C C C C C C C C C C C C C C C C C C C . . . . . . . . . . . . . . . . . . . . . . C . C C C C C . C C C C C C C C C    
263	C C C C C C C C C C C C C C C C C C C C C C C C C C C C C C . . . . . . . . . . . . . . . . . . . . . . C . C C C C C . C C C C C C C C C    
264	C C C C C C C C C C C C C C C C C C C C C C C C C C C C C C . . . . . . . . . . . . . . . . . . . . . . C . C C C C C . C C C C C C C C C    
265	C C C C C C C C C C C C C C C C C C C C C C C C C C C C C C . . . . . . . . . . . . . . . . . . . . . . C . C C C C C . C C C C C C C C C    
266	A A A A A A A A A A A A A A A A A A A A A A A A A A A A A A . . . . . . . . . . . . . . . . . . . . . . A . A A A A A . A A A A A A A A A    
267	C C C C C C C C C C C C C C C C C C C C C C C C C C C C C C . . . . . . . . . . . . . . . . . . . . . . C . C C C C C . C C C C C C C C C    
268	C C C C C C C C C C C C C C C C C C C C C C C C C C C C C C . . . . . . . . . . . . C . . . . . . . . . C . C C C C C . C C C C C C C C C    
269	C C C C C C C C C C C C C C C C C C C C C C C C C C C C C C . . . . . . . . . . . . C . . . . . . . . . C . C C C C C . C C C C C C C C C    
270	C C C C C C C C C C C C C C C C C C C C C C C C C C C C C C . . . . . . . . . . . C C . . . . . . . . . C . C C C C C . C C C C C C C C C    
271	C T T T T T T T T T T T T T T T T T T T T T T T T T T T T T . . . . . . . . . . . T T . . . . . . . . . T . T T T T T . T T T T T T T T T    
272	C C C C C C C C C C C C C C C C C C C C C C C C C C C C C C . . . . . . . . . . . C C . . . . . . . . . C . C C C C C . C C C C C C C C C    
273	G G G G G G G G G G G G G G G G G G G G G G G G G G G G G G . . . . . . . . . . . G G . . . . . . . G . G . G G G G G . G G G G G G G G G    
274	A A A A A A A A A A A A A A A A A A A A A A A A A A A A A A . . . . . . . . . . A A A . . . . . . . A A A . A A A A A . A A A A A A A A A    
275	G G G G G G G G G G G G G G G G G G G G G G G G G G G G G G . . . . . . . . . . G G G . . . . . . . G G G . G G G G G . G G G G G G G G G    
276	T T T T T T T T T T T T T T T T T T T T T T T T T T T T T T . . . . . . . . . . T T T . . . . . . . T T T . T T T T T . T T T T T T T T T    
277	G G G G G G G G G G G G G G G G G G G G G G G G G G G G G G . . . . . . . . . . G G G . . . . . . . G G G . G G G G G . G G G G G G G G G    
278	C C C C C C C C C C C C C C C C C C C C C C C C C C C C C C . . . . . . . . . . C C C . . . . . . . C C C . C C C C C . C C C C C C C C C    
279	C C C C C C C C C C C C C C C C C C C C C C C C C C C C C C . . . . . . . . . . C C C . . . . . . . C C C . C C C C C . C C C C C C C C C    
280	C C C C C C C C C C C C C C C C C C C C C C C C C C C C C C . . . . . . . . . . C C C . . . . . . . C C C . C C C C C . C C C C C C C C C    
281	A A A A A A A A A A A A A A A A A A A A A A A A A A A A A A . . . . . . . . . . A A A . . . . . . . A A A . A A A A A . A A A A A A A A A    
282	T T T T T T T T T T T T T T T T T T T T T T T T T T T T T T . . . . . . . . . . T T T . . . . . . . T T T . T T T T T . T T T T T T T T T    
283	G G G G G G G G G G G G G G G G G G G G G G G G G G G G G G . . . . . . . . . . G G G . . . . . . . G G G G G G G G G . G G G G G G G G G    
284	G G G G G G G G G G G G G G G G G G G G G G G G G G G G G G . . . . . . . . . . G G G . . . . . . . G G G G G G G G G . G G G G G G G G G    
285	A A A A A A A A A A A A A A A A A A A A A A A A A A A A A A . . . . . . . . . . A A A . . . . . A . A A A A A A A A A . A A A A A A A A A    
286	G G G G G G G G G G G G G G G G G G G G G G G G G G G G G G . . . . . . . . . . G G G . . . . . G . G G G G G G G G G . G G G G G G G G G    
287	A A A A A A A A A A A A A A A A A A A A A A A A A A A A A A . . . . . . . . . . A A A . . . . . A . A A A A A A A A A . A A A A A A A A A    
288	A A A A A A A A A A A A A A A A A A A A A A A A A A A A A A . . . . . . . . . . A A A . . . . . A . A A A A A A A A A . A A A A A A A A A    
289	A A A A A A A A A A A A A A A A A A A A A A A A A A A A A A . . . . . . . . . . A A A . . . . . A . A A A A A A A A A . A A A A A A A A A    
290	G G G G G G G G G G G G G G G G G G G G G G G G G G G G G G . . . . . . . . . . G G G . . . . . G . G G G G G G G G G . G G G G G G G G G    
291	T T T T T T T T T T T T T T T T T T T T T T T T T T T T T T . . . . . . . . . . T T T . . . . T T . T T T T T T T T T . T T T T T T T T T    
292	C C C C C C C C C C C C C C C C C C C C C C C C C C C C C C . . . . . . . . . . C C C . . . . C C . C C C C C C C C C . C C C C C C C C C    
293	C C C C C C C C C C C C C C C C C C C C C C C C C C C C C C . . . . . . . . . . C C C . . . . C C . C C C C C C C C C . C C C C C C C C C    
294	T T T T T T T T T T T T T T T T T T T T T T T T T T T T T T . . . . . . . . . . T T T . . . . T T T T T T T T T T T T . T T T T T T T T T    
295	T T T T T T T G T T T T T T T T T T T T T G T T T T T G T T . . . . . . . . . . T G T . . . . T T T T T T T T T T T T . T T T G G G G G T  9	 transversion  
296	T T T T T T T T T T T T T T T T T T T T T T T T T T T T T T . . . . . . . . . . T T T . . . . T T T T T T T T T T T T . T T T T T T T T T    
297	C C C C C C C C C C C C C C C C C C C C C C C C C C C C C C . . . . . . . . . . C C C . . . . C C C C C C C C C C C C . C C C C C C C C C    
298	A A A A A A A A A A A A A A A A A A A A A A A A A A A A A A . . . . . . . . . . A A A . . . . A A A A A A A A A A A A . A A A A A A A A A    
299	C C C C C C C C C C C C C C C C C C C C C C C C C C C C C C . . . . . . . . . . C C C . . . . C C C C C C C C C C C C . C C C C C C C C C    
300	T T T T T T T T T T T T T T T T T T T T T T T T T T T T T T . . . . . . . . . . T T T . . . . T T T T T T T T T T T T . T T T T T T T T T    
301	G G G G G G G G G G G G G G G G G G G G G G G G G G G G G G . . . . . . . . . . G G G . . . . G G G G G G G G G G G G . G G G G G G G G G    
302	C C C C C C C C C C C C C C C C C C C C C C C C C C C C C C . . . . . . . . . . C C C . . . . C C C C C C C C C C C C . C C C C C C C C C    
303	C C C C C C C C C C C C C C C C C C C C C C C C C C C C C C . . . . . . . . . . C C C . . . . C C C C C C C C C C C C . C C C C C C C C C    
304	A A A A A A A A A A A A A A A A A A A A A A A A A A A A A A . . . . . . . . . . A A A . . . . A A A A A A A A A A A A . A A A A A A A A A    
305	G G G G G G G G G G G G G G G G G G G G G G G G G G G G G G . . . . . . . . . . G G G . . . . G G G G G G G G G G G G . G G G G G G G G G    
306	A A A A A A A A A A A A A A A A A A A A A A A A A A A A A A . . . . . . . . . . A A A . . . . A A A A A A A A A A A A . A A A A A A A A A    
307	A A A A A A A A A A A A A A A A A A A A A A A A A A A A A A . . . . . . . . . . A A A . . . . A A A A A A A A A A A A . A A A A A A A A A    
308	C C C C C C C C C C C C C C C C C C C C C C C C C C C C C C . . . . . . . . . . C C C . . . . C C C C C C C C C C C C . C C C C C C C C C    
309	T T T T T T T T T T T T T T T T T T T T T T T T T T T T T T . . . . . . . . . . T T T . . . . T T T T T T T T T T T T . T T T T T T T T T    
310	C C C C C C C C C C C C C C C C C C C C C C C C C C C C C C . . . . . . . . . . C C C . . . . C C C C C C C C C C C C . C C C C C C C C C    
311	A A A A A A A A A A A A A A A A A A A A A A A A A A A A A A . . . . . . . . . . A A A . . . . A A A A A A A A A A A A . A A A A A A A A A    
312	A A A A A A A A A A A A A A A A A A A A A A A A A A A A A A . . . . . . . . . . A A A . . . . A A A A A A A A A A A A . A A A A A A A A A    
313	G G G G G G G G G G G G G G G G G G G G G G G G G G G G G G . . . . . . . . . . G G G . . . . G G G G G G G G G G G G . G G G G G G G G G    
314	T T T T T T T T T T T T T T T T T T T T T T T T T T T T T T . . . . . . . . . . T T T . . . . T T T T T T T T T T T T . T T T T T T T T T    
315	C C C C C C C C C C C C C C C C C C C C C C C C C C C C C C . . . . . . . . . . C C C . . . . C C C C C C C C C C C C . C C C C C C C C C    
316	C C C C C C C C C C C C C C C C C C C C C C C C C C C C C C . . . . . . . . . . C C C . . . . C C C C C C C C C C C C . C C C C C C C C C    
317	A A A A A A A A A A A A A A A A A A A A A A A A A A A A A A . . . . . . . . . . A A A . . . . A A A A A A A A A A A A . A A A A A A A A A    
318	A A A A A A A A A A A A A A A A A A A A A A A A A A A A A A . . . . . . . . . . A A A . . . . A A A A A A A A A A A A . A A A A A A A A A    
319	C C C C C C C C C C C C C C C C C C C C C C C C C C C C C C . . . . . . . . . . C C C . . . . C C C C C C C C C C C C . C C C C C C C C C    
320	C C C C C C C C C C C C C C C C C C C C C C C C C C C C C C . . . . . . . . . . C C C . . . . C C C C C C C C C C C C . C C C C C C C C C    
321	C C C C C C C C C C C C C C C C C C C C C C C C C C C C C C . . . . . . . . . . C C C . . . . C C C C C C C C C C C C . C C C C C C C C C    
322	T T T T T T T T T T T T T T T T T T T T T T T T T T T T T T . . . . . . . . . . T T T . . . . T T T T T T T T T T T T . T T T T T T T T T    
323	T T T T T T T T T T T T T T T T T T T T T T T T T T T T T T . . . . . . . . . . T T T . . . . T T T T T T T T T T T T . T T T T T T T T T    
324	T T T T T T T T T T T T T T T T T T T T T T T T T T T T T T . . . . . . . . . . T T T . . . . T T T T T T T T T T T T . T T T T T T T T T    
325	C C C C C C C C C C C C C C C C C C C C C C C C C C C C C C . . . . . . . . . . C C C . . . . C C C C C C C C C C C C . C C C C C C C C C    
326	A A A A A A A A A A A A A A A A A A A A A A A A A A A A A A . . . . . . . . . . A A A . . . . A A A A A A A A A A A A . A A A A A A A A A    
327	G G G G G G G G G G G G G - G G G G G G G G G G G G G G G G . . . . . . . . . . G G G . . . . G G G G G G G G G G G - . G G G G G G G G G    
328	A A A A A A A A A A A A A - A A A A A A A A A A A A A A A A . . . . . . . . . . A A A . . . . A A A A A A A A A A A - . A A A A A A A A A    
329	A A A A A A A A A A A A A A A A A A A A A A A A A A A A A A . . . . . . . . . . A A A . . . . A A A N A A A A A A A A . A A A A A A A A A    
330	A A A A A A A A A A A A A A A A A A A A A A A A A A A A A A . . . . . . . . . . A A A . . . . A A A A A A A A A A A A . A A A A A A A A A    
331	A A A A A A A A A A A A A A A A A A A A A A A A A A A A A A . . . . . . . . . . A A A . . . . A A A A A A A A A A A A . A A A A A A A A A    
332	A A A A A A A A A A A A A A A A A A A A A A A A A A A A A A . . . . . . . . . . A A A . . . . A A A A A A A A A A A A . A A A A A A A A A    
333	G G G G G G G G G G G G G G G G G G G G G G G G G G G G G G . . . . . . . . . . G G G . . . G G G G G G G G G G G G G . G G G G G G G G G    
334	G G G G G G G G G G G G G G G G G G G G G G G G G G G G G G . . . . . . . . . . G G G . . . G G G G G G G G G G G G G . G G G G G G G G G    
335	A A A A A A A A A A A A A A A A A A A A A A A A A A A A A A . . . . . . . . . . A A A . . . A A A A A A A A A A A A A . A A A A A A A A A    
336	T T T T T T T T T T T T T T T T T T T T T T T T T T T T T T . . . . . . . . . . T T T . . . T T T T T T T T T T T T T . T T T T T T T T T    
337	C C C C C C C C C C C C C C C C C C C C C C C C C C C C C C . . . . . . . . . . C C C . . . C C C C C C C C C C C C C . C C C C C C C C C    
338	T T T T T T T T T T T T T T T T T T T T T T T T T T T T T T . . . . . . . . . . T T T . . . T T T T T T T T T T T T T . T T T T T T T T T    
339	G G G G G G G G G G G G G G G G G G G G G G G G G G G G G G . . . . . . . . . . G G G . . . G G G G G G G G G G G G G . G G G G G G G G G    
340	T T T T T T T T T T T T T T T T T T T T T T T T T T T T T T . . . . . . . . . . T T T . . . T T T T T T T T T T T T T . T T T T T T T T T    
341	C C C C C C C C C C C C C C C C C C C C C C C C C C C C C C . . . . . . . . . . C C C . . . C C C C C C C C C C C C C . C C C C C C C C C    
342	A A A A A A A A A A A A A A A A A A A A A A A A A A A A A A . . . . . . . . . . A A A . . . A A A A A A A A A A A A A . A A A A A A A A A    
343	C C C C C C C C C C C C C C C C C C C C C C C C C C C C C C . . . . . . . . . . C C C . . . C C C C C C C C C C C C C . C C C C C C C C C    
344	G G G G G G G G G G G G G G G G G G G G G G G G G G G G G G . . . . . . . . . . G G G . . . G G G G G G G G G G G G G . G G G G G G G G G    
345	T T T T T T T T T T T T T T T T T T T T T T T T T T T T T T . . . . . . . . . . T T T . . . T T T T T T T T T T T T T . T T T T T T T T T    
346	A A A A A A A A A A A A A A A A A A A A A A A A A A A A A A . . . . . . . . . . A A A . . . A A A A A A A A A A A A A . A A A A A A A A A    
347	T T T T T T T T T T T T T T T T T T T T T T T T T T T T T T . . . . . . . . . . T T T . . . T T T T T T T T T T T T T . T T T T T T T T T    
348	T T T T T T T T T T T T T T T T T T T T T T T T T T T T T T . . . . . . . . . . T T T . . . T T T T T T T T T T T T T . T T T T T T T T T    
349	C C C C C C C C C C C C C C C C C C C C C C C C C C C C C C . . . . . . . . . . C C C . . . C C C C C C C C C C C C C . C C C C C C C C C    
350	T T T T T T T T T T T T T T T T T T T T T T T T T T T T T T . . . . . . . . . . T T T . . . T T T T T T T T T T T T T . T T T T T T T T T    
351	C C C C C C C C C C C C C C C C C C C C C C C C C C C C C C . . . . . . . . . . C C C . . . C C C C C C C C C C C C C . C C C C C C C C C    
352	A A A A A A A A A A A A A A A A A A A A A A A A A A A A A A . . . . . . . . . . A A A . . . A A A A A A A A A A A A A . A A A A A A A A A    
353	A A A A A A A A A A A A A A A A A A A A A A A A A A A A A A . . . . . . . . . . A A A . . . A A A A A A A A A A A A A . A A A A A A A A A    
354	C C C C C C C C C C C C C C C C C C C C C C C C C C C C C C . . . . . . . . . . C C C . . . C C C C C C C C C C C C C . C C C C C C C C C    
355	A A A A A A A A A A A A A A A A A A A A A A A A A A A A A A . . . . . . . . . . A A A . . . A A A A A A A A A A A A A . A A A A A A A A A    
356	T T T T T T T T T T T T T T T T T T T T T T T T T T T T T T . . . . . . . . . . T T T . . . T T T T T T T T T T T T T . T T T T T T T T T    
357	C C C C C C C C C C C C C C C C C C C C C C C C C C C C C C . . . . . . . . . . C C C . . . C C C C C C C C C C C C C . C C C C C C C C C    
358	T T T T T T T T T T T T T T T T T T T T T T T T T T T T T T . . . . . . . . . . T T T . . . T T T T T T T T T T T T T . T T T T T T T T T    
359	G G G G G G G G G G G G G G G G G G G G G G G G G G G G G G . . . . . . . . . G G G G . . . G G G G G G G G G G G G G . G G G G G G G G G    
360	A A A A A A A A A A A A A A A A A A A A A A A A A A A A A A . . . . . . . . . A A A A . . . A A A A A A A A A A A A A . A A A A A A A A A    
361	T T T T T T T T T T T T T T T T T T T T T T T T T T T T T T . . . . . . . . . T T T T . . . T T T T T T T T T T T T T . T T T T T T T T T    
362	A A A A A A A A A A A A A A A A A A A A A A A A A A A A A A . . . . . . . . . A A A A . . . A A A A A A A A A A A A A . A A A A A A A A A    
363	T T T T T T T T T T T T T T T T T T T T T T T T T T T T T T . . . . . . . . . T T T T . . . T T T T T T T T T T T T T . T T T T T T T T T    
364	G G G G G G G G G G G G G G G G G G G G G G G G G G G G G G . . . . . . . . . G G G G . . . G G G G G G G G G G G G G . G G G G G G G G G    
365	A A A A A A A A A A A A A A A A A A A A A A A A A A A A A A . . . . . . . . . A A A A . . . A A A A A A A A A A A A A . A A A A A A A A A    
366	A A A A A A A A A A A A A A A A A A A A A A A A A A A A A A . . . . . . . . . A A A A . . . A A A A A A A A A A A A A . A A A A A A A A A    
367	A A A A A A A A A A A A A A A A A A A A A A A A A A A A A A . . . . . . . . . A A A A . . . A A A A A A A A A A A A A . A A A A A A A A A    
368	G G G G G G G G G G G G G G G G G G G G G G G G G G G G G G . . . . . . . . . G G G G . . . G G G G G G G G G G G G G . G G G G G G G G G    
369	A A A A A A A A A A A A A A A A A A A A A A A A A A A A A A . . . . . . . . . A A A A . . . A A A A A A A A A A A A A . A A A A A A A A A    
370	T T T T T T T T T T T T T T T T T T T T T T T T T T T T T T . . . . . . . . . T T T T . . . T T T T T T T T T T T T T . T T T T T T T T T    
371	G G G G G G G G G G G G G G G G G G G G G G G G G G G G G G . . . . . . . G G G G G G . . . G G G G G G G G G G G G G . G G G G G G G G G    
372	G G G G G G G G G G G G G G G G G G G G G G G G G G G G G G . . . . . . . G G G G G G . . . G G G G G G G G G G G G G . G G G G G G G G G    
373	A A A A A A A A A A A A A A A A A A A A A A A A A A A A A A . . . . . . . A A A A A A . . . A A A A A A A A A A A A A . A A A A A A A A A    
374	A A A A A A A A A A A A A A A A A A A A A A A A A A A A A A . . . . . . . A A A A A A . . . A A A A A A A A A A A A A . A A A A A A A A A    
375	G G G G G G G G G G G G G G G G G G G G G G G G G G G G G G . . . . . . . G G G G G G . . . G G G G G G G G G G G G G . G G G G G G G G G    
376	T T T T T T T T T T T T T T T T T T T T T T T T T T T T T T . . . . . . . T T T T T T . . . T T T T T T T T T T T T T . T T T T T T T T T    
377	C C C C C C C C C C C C C C C C C C C C C C C C C C C C C C . . . . . . . C C C C C C . . . C C C C C C C C C C C C C . C C C C C C C C C    
378	T T T T T T T T T T T T T T T T T T T T T T T T T T T T T T . . . . . . . T T T T T T . . . T T T T T T T T T T T T T . T T T T T T T T T    
379	C C C C C C C C C C C C C C C C C C C C C C C C C C C C C C . . . . . . . C C C C C C . . . C C C C C C C C C C C C C . C C C C C C C C C    
380	A A A A A A A A A A A A A A A A A A A A A A A A A A A A A A . . . . . . . A A A A A A . . . A A A A A A A A A A A A A . A A A A A A A A A    
381	C C C C C C C C C C C C C C C C C C C C C C C C C C C C C C . . . . . . . C C C C C C . . . C C C C C C C C C C C C C . C C C C C C C C C    
382	C C C C C C C C C C C C C C C C C C C C C C C C C C C C C C . . . . . . . C C C C C C . . . C C C C C C C C C C C C C . C C C C C C C C C    
383	T T T T T T T T T T T T T T T T T T T T T T T T T T T T T T . . . . . . . T T T T T T . . . T T T T T T T T T T T T T . T T T T T T T T T    
384	T T T T T T T T T T T T T T T T T T T T T T T T T T T T T T . . . . . . . T T T T T T . . . T T T T T T T T T T T T T . T T T T T T T T T    
385	T T T T T T T T T T T T T T T T T T T T T T T T T T T T T T . . . . . . . T T T T T T . . . T T T T T T T T T T T T T . T T T T T T T T T    
386	G G G G G G G G G G G G G G G G G G G G G G G G G G G G G G . . . . . G G G G G G G G G . . G G G G G G G G G G G G G . G G G G G G G G G    
387	A A A A A A A A A A A A A A A A A A A A A A A A A A A A A A . . . . . A A A A A A A A A . . A A A A A A A A A A A A A . A A A A A A A A A    
388	G G G G G G G G G G G G G G G G G G G G G G G G G G G G G G . . . . . G G G G G G G G G . G G G G G G G G G G G G G G . G G G G G G G G G    
389	G G G G G G G G G G G G G G G G G G G G G G G G G G G G G G . . . . . G G G G G G G G G G G G G G G G G G G G G G G G . G G G G G G G G G    
390	A A A A A A A A A A A A A A A A A A A A A A A A A A A A A A . . . . . A A A A A A A A A A A A A A A A A A A A A A A A . A A A A A A A A A    
391	C C C C C C C C C C C C C C C C C C C C C C C C C C C C C C . . . . . C C C C C C C C C C C C C C C C C C C C C C C C . C C C C C C C C C    
392	T T T T T T T T T T T T T T T T T T T T T T T T T T T T T T . . . . . T T T T T T T T T T T T T T T T T T T T T T T T . T T T T T T T T T    
393	T T T T T T T T T T T T T T T T T T T T T T T T T T T T T T . . . . . T T T T T T T T T T T T T T T T T T T T T T T T . T T T T T T T T T    
394	C C C C C C C C C C C C C C C C C C C C C C C C C C C C C C . . . . . C C C C C C C C C C C C C C C C C C C C C C C C . C C C C C C C C C    
395	C C C C C C C C C C C C C C C C C C C C C C C C C C C C C C . . . . . C C C C C C C C C C C C C C C C C C C C C C C C . C C C C C C C C C    
396	T T T T T T T T T T T T T T T T T T T T T T T T T T T T T T . . . . . T T T T T T T T T T T T T T T T T T T T T T T T . T T T T T T T T T    
397	T T T T T T T T T T T T T T T T T T T T T T T T T T T T T T . . . . . T T T T T T T T T T T T T T T T T T T T T T T T . T T T T T T T T T    
398	G G G G G G G G G G G G G G G G G G G G G G G G G G G G G G . . . . . G G G G G G G G G G G G G G G G G G G G G G G G . G G G G G G G G G    
399	A A A A A A A A A A A A A A A A A A A A A A A A A A A A A A . . . . . A A A A A A A A A A A A A A A A A A A A A A A A . A A A A A A A A A    
400	T T T T T T T T T T T T T T T T T T T T T T T T T T T T T T . . . . . T T T T T T T T T T T T T T T T T T T T T T T T . T T T T T T T T T    
401	C C C C C C C C C C C C C C C C C C C C C C C C C C C C C C . . . . . C C C C C C C C C C C C C C C C C C C C C C C C . C C C C C C C C C    
402	T T T T T T T T T T T T T T T T T T T T T T T T T T T T T T . . . . . T T T T T T T T T T T T T T T T T T T T T T T T . T T T T T T T T T    
403	C C C C C C C C C C C C C T C C C C C C C C C C C C C C C C . . . . . C C C C C C C C C C C C C C C C C C C C C C C T . C C C C C C C C C    
404	T T T T T T T T T T T T T T T T T T T T T T T T T T T T T T . . . . . T T T T T T T T T T T T T T T T T T T T T T T T . T T T T T T T T T    
405	T T T T T T T T T T T T T T T T T T T T T T T T T T T T T T . . . . . T T T T T T T T T T T T T T T T T T T T T T T T . T T T T T T T T T    
406	G G G G G G G G G G G G G G G G G G G G G G G G G G G G G G . . . . . G G G G G G G G G G G G G G G G G G G G G G G G . G G G G G G G G G    
407	A A A A A A A A A A A A A A A A A A A A A A A A A A A A A A . . . . . A A A A A A A A A A A A A A A A A A A A A A A A . A A A A A A A A A    
408	G G G G G G G G G G G G G G G G G G G G G G G G G G G G G G . . . . . G G G G G G G G G G G G G G G G G G G G G G G G . G G G G G G G G G    
409	T T T T T T T T T T T T T T T T T T T T T T T T T T T T T T . . . . . T T T T T T T T T T T T T T T T T T T T T T T T . T T T T T T T T T    
410	G G G G G G G G G G G G G G G G G G G G G G G G G G G G G G . . . . . G G G G G G G G G G G G G G G G G G G G G G G G . G G G G G G G G G    
411	C C C C C C C C C C C C C C C C C C C C C C C C C C C C C C . . . . . C C C C C C C C C C C C C C C C C C C C C C C C . C C C C C C C C C    
412	C C C C C C C C C C C C C C C C C C C C C C C C C C C C C C . . . . . C C C C C C C C C C C C C C C C C C C C C C C C C C C C C C C C C C    
413	T T T T T T T T T T T T T T T T T T T T T T T T T T T T T T . . . . . T T T T T T T T T T T T T T T T T T T T T T T T T T T T T T T T T T    
414	T T T T T T T T T T T T T T T T T T T T T T T T T T T T T T . . . . . T T T T T T T T T T T T T T T T T T T T T T T T T T T T T T T T T T    
415	C C C C C C C C C C C C C C C C C C C C C C C C C C C C C C . . . . . C C C C C C C C C C C C C C C C C C C C C C C C C C C C C C C C C C    
416	A A A A A A A A A A A A A A A A A A A A A A A A A A A A A A . . . . . A A A A A A A A A A A A A A A A A A A A A A A A A A A A A A A A A A    
417	G G G G G G G G G G G G G G G G G G G G G G G G G G G G G G . . . . . G G G G G G G G G G G G G G G G G G G G G G G G G G G G G G G G G G    
418	T T T T T T T T T T T T T T T T T T T T T T T T T T T T T T . . . . . T T T T T T T T T T T T T T T T T T T T T T T T T T T T T T T T T T    
419	G G G G G G G G G G G G G G G G G G G G G G G G G G G G G G . . . . . G G G G G G G G G G G G G G G G G G G G G G G G G G G G G G G G G G    
420	A A A A A A A A A A A A A A A A A A A A A A A A A A A A A A . . . . . A A A A A A A A A A A A A A A A A A A A A A A A A A A A A A A A A A    
421	T T T T T T T T T T T T T T T T T T T T T T T T T T T T T T . . . . . T T T T T T T T T T T T T T T T T T T T T T T T T T T T T T T T T T    
422	T T T T T T T T T T T T T T T T T T T T T T T T T T T T T T . . . . . T T T T T T T T T T T T T T T T T T T T T T T T T T T T T T T T T T    
423	C C C C C C C C C C C C C C C C C C C C C C C C C C C C C C . . . . . C C C C C C C C C C C C C C C C C C C C C C C C C C C C C C C C C C    
424	A A A A A A A A A A A A A A A A A A A A A A A A A A A A A A . . . . . A A A A A A A A A A A A A A A A A A A A A A A A A A A A A A A A A A    
425	G G G G G G G G G G G G G G G G G G G G G G G G G G G G G G . . . . . G G G G G G G G G G G G G G G G G G G G G G G G G G G G G G G G G G    
426	C C C C C C C C C C C C C C C C C C C C C C C C C C C C C C . . . . . C C C C C C C C C C C C C C C C C C C C C C C C C C C C C C C C C C    
427	T T T T T T T T T T T T T T T T T T T T T T T T T T T T T T . . . . . T T T T T T T T T T T T T T T T T T T T T T T T T T T T T T T T T T    
428	A A A A A A A A A A A A A A A A A A A A A A A A A A A A A A . . . . . A A A A A A A A A A A A A A A A A A A A A A A A A A A A A A A A A A    
429	C C C C C C C C C C C C C C C C C C C C C C C C C C C C C C . . . . . C C C C C C C C C C C C C C C C C C C C C C C C C C C C C C C C C C    
430	A A A A A A A A A A A A A A A A A A A A A A A A A A A A A A . . . . . A A A A A A A A A A A A A A A A A A A A A A A A A A A A A A A A A A    
431	C C C C C C C C C C C C C C C C C C C C C C C C C C C C C C . . . . . C C C C C C C C C C C C C C C C C C C C C C C C C C C C C C C C C C    
432	T T T T T T T T T T T T T T T T T T T T T T T T T T T T T T . . . . . T T T T T T T T T T T T T T T T T T T T T T T T T T T T T T T T T T    
433	G G G G G G G G G G G G G G G G G G G G G G G G G G G G G G . . . . . G G G G G G G G G G G G G G G G G G G G G G G G G G G G G G G G G G    
434	G G G G G G G G G G G G G G G G G G G G G G G G G G G G G G . . . . . G G G G G G G G G G G G G G G G G G G G G G G G G G G G G G G G G G    
435	A A A A A A A A A A A A A A A A A A A A A A A A A A A A A A . . . . . A A A A A A A A A A A A A A A A A A A A A A A A A A A A A A A A A A    
436	A A A A A A A A A A A A A A A A A A A A A A A A A A A A A A . . . . . A A A A A A A A A A A A A A A A A A A A A A A A A A A A A A A A A A    
437	A A A A A A A A A A A A A A A A A A A A A A A A A A A A A A . . . . . A A A A A A A A A A A A A A A A A A A A A A A A A A A A A A A A A A    
438	T T T T T T T T T T T T T T T T T T T T T T T T T T T T T T . . . . . T T T T T T T T T T T T T T T T T T T T T T T T T T T T T T T T T T    
439	C C C C C C C C C C C C C C C C C C C C C C C C C C C C C C . . . . . C C C C C C C C C C C C C C C C C C C C C C C C C C C C C C C C C C    
440	A A A A A A A A A A A A A A A A A A A A A A A A A A A G A A . . . . . A A A A A A A A A A A A A A A A A A A A A A A A A A A A A A A A A A    
441	A A A A A A A A A A A A A A A A A A A A A A A A A A A A A A . . . . . A A A A A A A A A A A A A A A A A A A A A A A A A A A A A A A A A A    
442	G G G G G G G G G G G G G G G G G G G G G G G G G G G G G G . . . . . G G G G G G G G G G G G G G G G G G G G G G G G G G G G G G G G G G    
443	T T T T T T T T T T T T T T T T T T T T T T T T T T T T T T . . . . . T T T T T T T T T T T T T T T T T T T T T T T T T T T T T T T T T T    
444	- - - - - - - - - - - - - - - - - - - - - - - - - - - - - - . . . . . - - - - T - - - - - - - - - - - - - - - - - - - - - - - - - - - - -    
445	C C C C C C C C C C C C C C C C C C C C C C C C C C C C C C . . . . . C C C C C C C C C C C C C C C C C C C C C C C C C C C C C C C C C C    
446	C C C C C C C C C C C C C C C C C C C C C C C C C C C C C C . . . . . C C C C C C C C C C C C C C C C C C C C C C C C C C C C C C C C C C    
447	C C C C C C C C C C C C C C C C C C C C C C C C C C C C C C . . . . . C C C C C C C C C C C C C C C C C C C C C C C C C C C C C C C C C C    
448	A A A A A A A A A A A A A A A A A A A A A A A A A A A A A A . . . . . A A A A A A A A A A A A A A A A A A A A A C A A A A A A A A A A A A    
449	C C C C C C C C C C C C C C C C C C C C C C C C C C C C C C . . . . . C C C C C C C C C C C C C C C C C C C C C C C C C C C C C C C C C C    
450	T T T T T T T T T T T T T T T T T T T T T T T T T T T T T T . . . . . T T T T T T T T T T T T T T T T T T T T T T T T T T T T T T T T T T    
451	A A A A A A A A A A A A A A A A A A A A A A A A A A A A A A . . . . . G G A A A A A A A A A A A A A A A A A A A A A A A A A A A A A A A A    
452	C C C C C C C C C C C C C C C C C C C C C C C C C C C C C C . . . . . C C C C C C C C C C C C C C C C C C C C C C C C C C C C C C C C C C    
453	G G G G G G G G G G G G G G G G G G G G G G G G G G G G G G . . . . . G G G G G G G G G G G G G G G G G G G G G G G G G G G G G G G G G G    
454	C C C C C C C C C C C C C C C C C C C C C C C C C C C C C C . . . . . C C C C C C C C C C C C C C C C C C C C C C C C C C C C C C C C C C    
455	A A A A A A A A A A A A A A A A A A A A A A A A A A A A A A . . . . . A A A A A A A A A A A A A A A A A A A A A A A A A A A A A A A A A A    
456	T T T T T T T T T T T T T T T T T T T T T T T T T T T T T T . . . . . T T T T T T T T T T T T T T T T T T T T T T T T T T T T T T T T T T    
457	T T T T T T T T T T T T T T T T T T T T T T T T T T T T T T . . . . . T T T T T T T T T T T T T T T T T T T T T T T T T T T T T T T T T T    
458	C C C C C C C C C C C C C C C C C C C C C C C C C C C C C C . . . . . C C C C C C C C C C C C C C C C C C C C C C C C C C C C C C C C C C    
459	C C C C C C C C C C C C C C C C C C C C C C C C C C C C C C . . . . . C C C C C C C C C C C C C C C C C C C C C C C C C C C C C C C C C C    
460	G G G G G G G G G G G G G G G G G G G G G G G G G G G G G G . . . . . G G G G G G G G G G G G G G G G G G G G G G G G G G G G G G G G G G    
461	C C C C C C C C C C C C C C C C C C C C C C C C C C C C C C . . . . . C C C C C C C C C C C C C C C C C C C C C C C C C C C C C C C C C C    
462	- - - - - - - - - - - - - - - - - - - - - - - - - - - - - - . . . . . - - - - C - - - - - - - - - - - - - - - - - - - - - - - - - - - - -    
463	A A A A A A A A A A A A A A A A A A A A A A A A A A A A A A . . . . . A A A A A A A A A A A A A A A A A A A A A A A A A A A A A A A A A A    
464	T T T T T T T T T T T T T T T T T T T T T T T T T T T T T T . . . . . T T T T T T T T T T T T T T T T T T T T T T T T T T T T T T T T T T    
465	T T T T T T T T T T T T T T T T T T T T T T T T T T T T T T . . . . . T T T T T T T T T T T T T T T T T T T T T T T T T T T T T T T T T T    
466	T T T T T T T T T T T T T T T T T T T T T T T T T T T T T T . . . . . T T T T T T T T T T T T T T T T T T T T T T T T T T T T T T T T T T    
467	T T T T T T T T T T T T T T T T T T T T T T T T T T T T T T . . . . . T T T T T T T T T T T T T T T T T T T T T T T T T T T T T T T T T T    
468	T T T T T T T T T T T T T T T T T T T T T T T T T T T T T T . . . . . T T T T T T T T T T T T T T T T T T T T T T T T T T T T T T T T T T    
469	G G G G G G G G G G G G G G G G G G G G G G G G G G G G G G . . . . . G G G G G G G G G G G G G G G G G G G G G G G G G G G G G G G G G G    
470	A A A A A A A A A A A A A A A A A A A A A A A A A A A A A A . . . . . A A A A A A A A A A A A A A A A A A A A A A A A A A A A A A A A A A    
471	C C C C C C C C C C C C C C C C C C C C C C C C C C C C C C . . . . . C C C C C C C C C C C C C C C C C C C C C C C C C C C C C C C C C C    
472	T T T T T T T T T T T T T T T T T T T T T T T T T T T T T T . . . . . T T T T T T T T T T T T T T T T T T T T T T T T T T T T T T T T T T    
473	T T T T T T T T T T T T T T T T T T T T T T T T T T T T T T . . . . . T T T T T T T T T T T T T T T T T T T T T T T T T T T T T T T T T T    
474	T T T T T T T T T T T T T T T T T T T T T T T T T T T T T T . . . . . T T T T T T T T T T T T T T T T T T T T T T T T T T T T T T T T T T    
475	G G G G G G G G G G G G G G G G G G G G G G G G G G G G G G . . . . . G G G G G G G G G G G G G G G G G G G G G G G G G G G G G G G G G G    
476	A A A A A A A A A A A A A A A A A A A A A A A A A A A A A A . . . . . A A A A A A A A A A A A A A A A A A A A A A A A A A A A A A A A A A    
477	T T T T T T T T T T T T T T T T T T T T T T T T T T T T T T . . . . . T T T T T T T T T T T T T T T T T T T T T T T T T T T T T T T T T T    
478	G G G G G G G G G G G G G G G G G G G G G G G G G G G G G G . . . . . G G G G G G G G G G G G G G G G G G G G G G G G G G G G G G G G G G    
479	A A A A A A A A A A A A A A A A A A A A A A A A A A A A A A . . . . . A A A A A A A A A A A A A A A A A A A A A A A A A A A A A A A A A A    
480	T T T T T T T T T T T T T T T T T T T T T T T T T T T T T T . . . . . T T T T T T T T T T T T T T T T T T T T T T T T T T T T T T T T T T    
481	G G G G G G G G G G G G G G G G G G G G G G G G G G G G G G . . . . . G G G G G G G G G G G G G G G G G G G G G G G G G G G G G G G G G G    
482	A A A A A A A A A A A A A A A A A A A A A A A A A A A A A A . . . . . A A A A A A A A A A A A A A A A A A A A A A A A A A A A A A A A A A    
483	T T T T T T T T T T T T T T T T T T T T T T T T T T T T T T . . . . . T T T T T T T T T T T T T T T T T T T T T T T T T T T T T T T T T T    
484	G G G G G G G G G G G G G G G G G G G G G G G G G G G G G G . . . . . G G G G G G G G G G G G G G G G G G G G G G G G G G G G G G G G G G    
485	G G G G G G G G G G G G G G G G G G G G G G G G G G G G G G . . . . . G G G G G G G G G G G G G G G G G G G G G G G G G G G G G G G G G G    
486	C C C C C A C C C C C C C C C C C C C C C C C C C C C C C C . . . . . C C C C C C C C C C C C C C C C C C C C C C C C C C C C C C C C C C    
487	A A A A A C A A A A A A A A A A A A A A A A A A A A A A A A . . . . . A A A A A A A A A A A A A A A A A A A A A A A A A A A A A A A A A A    
488	C C C C C C C C C C C C C C C C C C C C C C C C C C C C C C . . . . . C C C C C C C C C C C C C C C C C C C C C C C C C C C C C C C C C C    
489	T T T T T T T T T T T T T T T T T T T T T T T T T T T T T T . . . . . T T T T T T T T T T T T T T T T T T T T T T T T T T T T T T T T T T    
490	C C C C C C C C C C C C C C C C C C C C C C C C C C C C C C . . . . . C C C C C C C C C C C C C C C C C C C C C C C C C C C C C C C C C C    
491	T T T T T T T T T T T T T T T T T T T T T T T T T T T T T T . . . . . T T T T T T T T T T T T T T T T T T T T T T T T T T T T T T T T T T    
492	G G G G G G G G G G G G G G G G G G G G G G G G G G G G G G . . . . . G G G G G G G G G G G G G G G G G G G G G G G G G G G G G G G G G G    
493	G G G G G G G G G G G G G G G G G G G G G G G G G G G G G G . . . . . G G G G G G G G G G G G G G G G G G G G G G G G G G G G G G G G G G    
494	A A A A A A A A A A A A A A A A A A A A A A A A A A A A A A . . . . . A A A A A A A A A A A A A A A A A A A A A A A A A A A A A A A A A A    
495	T T T T T T T T T T T T T T T T T T T T T T T T T T T T T T . . . . . T T T T T T T T T T T T T T T T T T T T T T T T T T T T T T T T T T    
496	. G G G G G G G G G G G G G G G G G G G G G G G G G G G G G . . . . . G G G G G G G G G G G G G G G G G G G G G G G G G G G G G G G G G G    
497	. G G G G G G G G G G G G G G G G G G G G G G G G G G G G G . . . . . G G G G G G G G G G G G G G G G G G G G G G G G G G G G G G G G G G    
498	. T T T T T T T T T T T T T T T T T T T T T T T T T T T T T . . . . . T T T T T T T T T T T T T T T T T T T T T T T T T T T T T T T T T T    
499	. G G G G G G G G G G G G G G G G G G G G G G G G G G G G G . . . . . G G G G G G G G G G G G G G G G G G G G G G G G G G G G G G G G G G    
500	. G G G G G G G G G G G G G G G G G G G G G G G G G G G G G . . . . . G G G G G G G G G G G G G G G G G G G G G G G G G G G G G G G G G G    
501	. G G G G G G G G G G G G G G G G G G G G G G G G G G G G G . . . . . G G G G G G G G G G G G G G G G G G G G G G G G G G G G G G G G G G    
502	. G G G G G G G G G G G G G G G G G G G G G G G G G G G G G . . . . . G G G G G G G G G G G G G G G G G G G G G G G G G G G G G G G G G G    
503	. A A A A A A A A A A A A A A A A A A A A A A A A A A A A A . . . . . A A A A A A A A A A A A A A A A A A A A A A A A A A A A A A A A A A    
504	. C C C C C C C C C C C C C C C C C C C C C C C C C C C C C . . . . . C C C C C C C C C C C C C C C C C C C C C C C C C C C C C C C C C C    
505	. C C C C C C C C C C C C C C C C C C C C C C C C C C C C C . . . . . C C C C C C C C C C C C C C C C C C C C C C C C C C C C C C C C C C    
506	. T T T T T T T T T T T T T T T T T T T T T T T T T T T T T . . . . . T T T T T T T T T T T T T T T T T T T T T T T T T T T T T T T T T T    
507	. G G G G G G G G G G G G G G G G G G G G G G G G G G G G G . . . . . G G G G G G G G G G G G G G G G G G G G G G G G G G G G G G G G G G    
508	. G G G G G G G G G G G G G G G G G G G G G G G G G G G G G . . . . . G G G G G G G G G G G G G G G G G G G G G G G G G G G G G G G G G G    
509	. A A A A A A A A A A A A A A A A A A A A A A A A A A A A A . . . . . A A A A A A A A A A A A A A A A A A A A A A A A A A A A A A A A A A    
510	. G G G G G G G G G G G G G G G G G G G G G G G G G G G G G . . . . . G G G G G G G G G G G G G G G G G G G G G G G G G G G G G G G G G G    
511	. A A A A A A A A A A A A A A A A A A A A A A A A A A A A A . . . . . A A A A A A A A A A A A A A A A A A A A A A A A A A A A A A A A A A    
512	. A A A A A A A A A A A A A A A A A A A A A A A A A A A A A . . . . . A A A A A A A A A A A A A A A A A A A A A A A A A A A A A A A A A A    
513	. G G G G G G G G G G G G G G G G G G G G G G G G G G G G G . . . . . G G G G G G G G G G G G G G G G G G G G G G G G G G G G G G G G G G    
514	. C C C C C C C C C C C C C C C C C C C C C T C C C C C C C . . . . . C C C C C C C C C C C C C C C C C C C C C C C C C C C C C C C C C T    
515	. T T T T T T T T T T T T T T T T T T T T T T T T T T T T T . . . . . T T T T T T T T T T T T T T T T T T T T T T T T T T T T T T T T T T    
516	. G G G G G G G G G G G G G G G G G G G G G G G G G G G G G . . . . . G G G G G G G G G G G G G G G G G G G G G G G G G G G G G G G G G G    
517	. G G G G G G G G G G G G G G G G G G G G G G G G G G G G G . . . . . G G G G G G G G G G G G G G G G G G G G G G G G G G G G G G G G G G    
518	. T T T T T T T T T T T T T T T T T T T T T T T T T T T T T . . . . . T T T T T T T T T T T T T T T T T T T T T T T T T T T T T T T T T T    
519	. G G G G G G G G G G G G G G G G G G G G G G G G G G G G G . . . . . G G G G G G G G G G G G G G G G G G G G G G G G G G G G G G G G G G    
520	. A A A A A A A A A A A A A A A A A A A A A A A A A A A A A . . . . . A A A A A A A A A A A A A A A A A A A A A A A A A A A A A A A A A A    
521	. A A A A A A A A A A A A A A A A A A A A A A A A A A A A A . . . . . A A A A A A A A A A A A A A A A A A A A A A A A A A A A A A A A A A    
522	. C C C C C C C C C C C C C C C C C C C C C C C C C C C C C . . . . . C C C C C C C C C C C C C C C C C C C C C C C C C C C C C C C C C C    
523	. T T T T T T T T T T T T T T T T T T T T T T T T T T T T T . . . . . T T T T T T T T T T T T T T T T T T T T T T T T T T T T T T T T T T    
524	. G G G G G G G G G G G G G G G G G G G G G G G G G G G G G . . . . . G G G G G G G G G G G G G G G G G G G G G G G G G G G G G G G G G G    
525	. T T T T T T T T T T T T T T T T T T T T T T T T T T T T T . . . . . T T T T T T T T T T T T T T T T T T T T T T T T T T T T T T T T T T    
526	. C C C C C C C C C C C C C C C C C C C C C C C C C C C C C . . . C C C C C C C C C C C C C C C C C C C C C C C C C C C C C C C C C C C C    
527	. T T T T T T T T T T T T T T T T T T T T T T T T T T T T T . . . T T T T T T T T T T T T T T T T T T T T T T T T T T T T T T T T T T T T    
528	. G G G G G G G G G G G G G G G G G G G G G G G G G G G G G . . . G G G G G G G G G G G G G G G G G G G G G G G G G G G G G G G G G G G G    
529	. A A A A A A A A A A A A A A A A A A A A A A A A A A A A A . . . A A A A A A A A A A A A A A A A A A A A A A A A A A A A A A A A A A A A    
530	. C C C C C C C C C C C C C C C C C C C C C C C C C C C C C . . . C C C C C C C C C C C C C C C C C C C C C C C C C C C C C C C C C C C C    
531	. T T T T T T T T T T T T T T T T T T T T T T T T T T T T T . . . T T T T T T T T T T T T T T T T T T T T T T T T T T T T T T T T T T T T    
532	. G G G G G G G G G G G G G G G G G G G G G G G G G G G G G . . . G G G G G G G G G G G G G G G G G G G G G G G G G G G G G G G G G G G G    
533	. G G G G G G G G G G G G G G G G G G G G G G G G G G G G G . . . G G G G G G G G G G G G G G G G G G G G G G G G G G G G G G G G G G G G    
534	. T T T T T T T T T T T T T T T T T T T T T T T T T T T T T . . . T T T T T T T T T T T T T T T T T T T T T T T T T T T T T T T T T T T T    
535	. G G G G G G G G G G G G G G G G G G G G G G G G G G G G G . . . G G G G G G G G G G G G G G G G G G G G G G G G G G G G G G G G G G G G    
536	. A A A A A A A A A A A A A A A A A A A A A A A A A A A A A . . . A A A A A A A A A A A A A A A A A A A A A A A A A A A A A A A A A A A A    
537	. G G G G G G G G G G G A G G G A G G G A G G G G G G G A G . . . G G G G G G G G G G G G G G G G G G G A A A G G G G G G G G G G G G G G  7	 transition  
538	. A A A A A A A A A A A A A A A A A A A A A A A A A A A A A . . . A A A A A A A A A A A A A A A A A A A A A A A A A A A A A A A A A A A A    
539	. C C C C C C C C C C C C C C C C C C C C C C C C C C C C C . . . C C C C C C C C C C C C C C C C C C C C C C C C C C C C C C C C C C C C    
540	. G G G G G G G G G G G G G G G G G G G G G G G G G G G G G . . . G G G G G G G G G G G G G G G G G G G G G G G G G G G G G G G G G G G G    
541	. G G G G G G G G G G G G G G G G G G G G G G G G G G G G G . . . G G G G G G G G G G G G G G G G G G G G G G G G G G G G G G G G G G G G    
542	. A A A A A A A A A A A A A A A A A A A A A A A A A A A A A . . . A A A A A A A A A A A A A A A A A A A A A A A A A A A A A A A A A A A A    
543	. T T T T T T T T T T T T T T T T T T T T T T T T T T T T T . . . T T T T T T T T T T T T T T T T T T T T T T T T T T T T T T T T T T T T    
544	. G G G G G G G G G G G G G G G G G G G G G G G G G G G G G . . . G G G G G G G G G G G G G G G G G G G G G G G G G G G G G G G G G G G G    
545	. A A A A A A A A A A A A A A A A A A A A G A A A A A A A A . . . A A A A A A A A A A A A A A A A A A A A A A A A A A A A A A G A A A A A    
546	. C C C C C C C C C C C C C C C C C C C C C C C C C C C C C . . . C C C C C C C C C C C C C C C C C C C C C C C C C C C C C C C C C C C C    
547	. A A A A A A A A A A A A A A A A A A A A A A A A A A A A A . . . A A A A A A A A A A A A A A A A A A A A A A A A A A A A A A A A A A A A    
548	. C C C C C C C C C C C C C C C C C C C C C C C C C C C C C . . . C C C C C C C C C C C C C C C C C C C C C C C C C C C C C C C C C C C C    
549	. A A A A A A A A A A A A A A A A A A A A A A A A A A A A A . . . A A A A A A A A A A A A A A A A A A A A A A A A A A A A A A A A A A A A    
550	. A A A A A A A A A A A A A A A A A A A A A A A A A A A A A . . . A A A A A A A A A A A A A A A A A A A A A A A A A A A A A A A A A A A A    
551	. G G G G G G G G G G G G G G G G G G G G G G G G G G G G G . . . G G G G G G G G G G G G G G G G G G G G G G G G G G G G G G G G G G G G    
552	. G G G G G G G G G G G G G G G G G G G G G G G G G G G G G . . . G G G G G G G G G G G G G G G G G G G G G G G G G G G G G G G G G G G G    
553	. C C C C C C C C C C C C C C C C C C C C C C C C C C C C C . . . C C C C C C C C C C C C C C C C C C C C C C C C C C C C C C C C C C C C    
554	. T T T T T T T T T T T T T T T T T T T T T T T T T T T T T . . . T T T T T T T T T T T T T T T T T T T T T T T T T T T T T T T T T T T T    
555	. T T T T T T T T T T T T T T T T T T T T T T T T T T T T T . . . T T T T T T T T T T T T T T T T T T T T T T T T T T T T T T T T T T T T    
556	. A A A A A A A A A A A A A A A A A A A A A A A A A A A A A . . . A A A A A A A A A A A A A A A A A A A A A A A A A A A A A A A A A A A A    
557	. C C C C C C C C C C C C C C C C C C C C C C C C C C C C C . . . C C C C C C C C C C C C C C C C C C C C C C C C C C C C C C C C C C C C    
558	. C C C C C C C C C C C C C C C C C C C C C C C C C C C C C . . . C C C C C C C C C C C C C C C C C C C C C C C C C C C C C C C C C C C C    
559	. A A A A A A A A A A A A A A A A A A A A A A A A A A A A A . . . A A A A A A A A A A A A A A A A A A A A A A A A A A A A A A A A A A A A    
560	. C C C C C C C C C C C C C C C C C C C C C C C C C C C C C . . . C C C C C C C C C C C C C C C C C C C C C C C C C C C C C C C C C C C C    
561	. T T T T T T T T T T T T T T T T T T T T T T T T T T T T T . . . T T T T T T T T T T T T T T T T T T T T T T T T T T T T T T T T T T T T    
562	. G G G G G G G G G G G G G G G G G G G G G G G G G G G G G . . . G G G G G G G G G G G G G G G G G G G G G G G G G G G G G G G G G G G G    
563	. A A A A A A A A A A A A A A A A A A A A A A A A A A A A A . . . A A A A A A A A A A A A A A A A A A A A A A A A A A A A A A A A A A A A    
564	. A A A A A A A A A A A A A A A A A A A A A A A A A A A A A . . . A A A A A A A A A A A A A A A A A A A A A A A A A A A A A A A A A A A A    
565	. G G G G G G G G G G G G G G G G G G G G G G G G G G G G G . . . G G G G G G G G G G G G G G G G G G G G G G G G G G G G G G G G G G G G    
566	. A A A A A A A A A A A A A A A A A A A A A A A A A A A A A . . . A A A A A A A A A A A A A A A A A A A A A A A A A A A A A A A A A A A A    
567	. G G G G G G G G G G G G G G G G G G G G G G G G G G G G G . . . G G G G G G G G G G G G G G G G G G G G G G G G G G G G G G G G G G G G    
568	. A A A A A A A A A A A A A A A A A A A A A A A A A A A A A . . . A A A A A A A A A A A A A A A A A A A A A A A A A A A A A A A A A A A A    
569	. T T T T T T T T T T T T T T T T T T T T T T T T T T T T T . . . T T T T T T T T T T T T T T T T T T T T T T T T T T T T T T T T T T T T    
570	. G G G G G G G G G G G G G G G G G G G G G G G G G G G G G . . . G G G G G G G G G G G G G G G G G G G G G G G G G G G G G G G G G G G G    
571	. A A A A A A A A A A A A A A A A A A A A A A A A A A A A A . . . A A A A A A A A A A A A A A A A A A A A A A A A A A A A A A A A A A A A    
572	. G G G G G G G G G G G G G G G G G G G G G G G G G G G G G . . . G G G G G G G G G G G G G G G G G G G G G G G G G G G G G G G G G G G G    
573	. G G G G G G G G G G G G G G G G G G G G G G G G G G G G G . . . G G G G G G G G G G G G G G G G G G G G G G G G G G G G G G G G G G G G    
574	. C C C C C C C C C C C C C C C C C C C C C C C C C C C C C . . . C C C C C C C C C C C C C C C C C C C C C C C C C C C C C C C C C C C C    
575	. A A A A A A A A A A A A A A A A A A A A A A A A A A A A A . . . A A A A A A A A A A A A A A A A A A A A A A A A A A A A A A A A A A A A    
576	. G G G G G G G G G G G G G G G G G G G G G G G G G G G G G . . . G G G G G G G G G G G G G G G G G G G G G G G G G G G G G G G G G G G G    
577	. C C C C C C C C C C C C C C C C C C C C C C C C C C C C C . . . C C C C C C C C C C C C C C C C C C C C C C C C C C C C C C C C C C C C    
578	. T T T T T T T T T T T T T T T T T T T T T T T T T T T T T . . . T T T T T T T T T T T T T T T T T T T T T T T T T T T T T T T T T T T T    
579	. C C C C C C C C C C C C C C C C C C C C C C C C C C C C C . . . C C C C C C C C C C C C C C C C C C C C C C C C C C C C C C C C C C C C    
580	. A A A A A A A A A A A A A A A A A A A A A A A A A A A A A . . . A A A A A A A A A A A A A A A A A A A A A A A A A A A A A A A A A A A A    
581	. T T T T T T T T T T T T T T T T T T T T T T T T T T T T T . . . T T T T T T T T T T T T T T T T T T T T T T T T T T T T T T T T T T T T    
582	. C C C C C C C C C C C C C C C C C C C C C C C C C C C C C . . . C C C C C C C C C C C C C C C C C C C C C C C C C C C C C C C C C C C C    
583	. . A A A A A A A A A A A A A A A A A A A A A A A A A A A A . . . A A A A A A A A A A A A A A A A A A A A A A A A A A A A A A A A A A A A    
584	. . G G G G G G G G G G G G G G G G G G G G G G G G G G G G . . . G G G G G G G G G G G G G G G G G G G G G G G G G G G G G G G G G G G G    
585	. . C C C C C C C C C C C C C C C C C C C C C C C C C C C C . . . C C C C C C C C C C C C C C C C C C C C C C C C C C C C C C C C C C C C    
586	. . A A A A A A A A A A A A A A A A A A A A A A A A A A A A . . . A A A A A A A A A A A A A A A A A A A A A A A A A A A A A A A A A A A A    
587	. . A A A A A A A A A A A A A A A A A A A A A A A A A A A A . . . A A A A A A A A A A A A A A A A A A A A A A A A A A A A A A A A A A A A    
588	. . T T T T T T T T T T T T T T T T T T T T T T T T T T T T . . . T T T T T T T T T T T T T T T T T T T T T T T T T T T T T T T T T T T T    
589	. . A A A A N A A A A A A A A A A A A A A A A A A A A A A A . . . A A A A A A A A A A A A A A A A A A A A A A A A A A A A A A A A A A A A    
590	. . - - N - A - - - - - - - - - - - - - - - - - - - - - - - . A . - - - - - - - - - - - - - - - - - - - - - - - - - - - - - - - - - - - -    
591	. . T T T T T T T T T T T T T T T T T T T T T T T T T T T T . T . T T T T T T T T T T T T T T T T T T T T T T T T T T T T T T T T T T T T    
592	. . T T T T T T T T T T T T T T T T T T T T T T T T T T T T . T . T T T T T T T T T T T T T T T T T T T T T T T T T T T T T T T T T T T T    
593	. . C C C C C C C C C C C C C C C C C C C C C C C C C C C C . C . C C C C C C C C C C C C C C C C C C C C C C C C C C C C C C C C C C C C    
594	. . T T T T T T T T T T T T T T T T T T T T T T T T T T T T . T . T T T T T T T T T T T T T T T T T T T T T T T T T T T T T T T T T T T T    
595	. . T T T T T T T T T T T T T T T T T T T T T T T T T T T C . T . T T T T T T T T T T T T T T T T T T T T T T T T T T T T T T T T T T T T    
596	. . G G G G G G G G G G G G G G G G G G G G G G G G G G G G . G . G G G G G G G G G G G G G G G G G G G G G G G G G G G G G G G G G G G G    
597	. . A A A A A A A A A A A A A A A A A A A A A A A A A A A A . A . A A A A A A A A A A A A A A A A A A A A A A A A A A A A A A A A A A A A    
598	. . A A A A A A A A A A A A A A A A A A A A A A A A A A A A . A . A A A A A A A A A A A A A A A A A A A A A A A A A A A A A A A A A A A A    
599	. . G G G G G G G G G G G G G G G G G G G G G G G G G G G G . G . G G G G G G G G G G G G G G G G G G G G G G G G G G G G G G G G G G G G    
600	. . A A A A A A A A A A A A A A A A A A A A A A A A A A A A . A . A A A A A A A A A A A A A A A A A A A A A A A A A A A A A A A A A A A A    
601	. . G G G G G G G G G G G G G G G G G G G G G G G G G G G G . G . G G G G G G G G G G G G G G G G G G G G G G G G G G G G G G G G G G G G    
602	. . T T T T T T T T T T T T T T T T T T T T T T T T T T T T . T . T T T T T T T T T T T T T T T T T T T T T T T T T T T T T T T T T T T T    
603	. . C C C C C C C C C C C C C C C C C C C C C C C C C C C C . C . C C C C C C C C C C C C C C C C C C C C C C C T C C T C C C C C C C C C    
604	. . T T T T T T T T T T T T T T T T T T T T T T T T T T T T . T T T T T T T T T T T T T T T T T T T T T T T T T T T T T T T T T T T T T T    
605	. . G G G G G G G G G G G G G G G G G G G G G G G G G G G G . G G G G G G G G G G G G G G G G G G G G G G G G G G G G G G G G G G G G G G    
606	. . A A A A A A A A A A A A A A A A A A A A A A A A A A A A . A A A A A A A A A A A A A A A A A A A A A A A A A A A A A A A A A A A A A A    
607	. . T T T T T T T T T T T T T T T T T T T T T T T T T T T T . T T T T T T T T T T T T T T T T T T T T T T T T T T T T T T T T T T T T T T    
608	. . A A A A A A A A A A A A A A A A A A A A A A A A A A A A . A A A A A A A A A A A A A A A A A A A A A A A A A A A A A A A A A A A A A A    
609	. . T T T T T T T T T T T T T T T T T T T T T T T T T T T T . T T T T T T T T T T T T T T T T T T T T T T T T T T T T T T T T T T T T T T    
610	. . A A A A A A A A A A A A A A A A A A A A A A A A A A A A . A A A A A A A A A A A A A A A A A A A A A A A A A A A A A A A A A A A A A A    
611	. . G G G G G G G G G G G G G G G G G G G G G G G G G G G G . G G G G G G G G G G G G G G G G G G G G G G G G G G G G G G G G G G G G G G    
612	. . A A A A A A A A A A A A A A A A A A A A A A A A A A A A . A A A A A A A A A A A A A A A A A A A A A A A A A A A A A A A A A A A A A A    
613	. . C C C C C C C C C C C C C C C C C C C C C C C C C C C C . C C C C C C C C C C C C C C C C C C C C C C C C C C C C C C C C C C C C C C    
614	. . A A A A A A A A A A A A A A A A A A A A A A A A A A A A . A A A A A A A A A A A A A A A A A A A A A A A A A A A A A A A A A A A A A A    
615	. . A A A A A A A A A A A A A A A A A A A A A A A A A A A A . A A A A A A A A A A A A A A A A A A A A A A A A A A A A A A A A A A A A A A    
616	. . A A A A A A A A A A A A A A A A A A A A A A A A A A A A . A A A A A A A A A A A A A A A A A A A A A A A A A A A A A A A A A A A A A A    
617	. . G G G G G G G G G G G G G G G G G G G G G G G G G G G G . G G G G G G G G G G G G G G G G G G G G G G G G G G G G G G G G G G G G G G    
618	. . A A A A A A A A A A A A A A A A A A A A A A A A A A A A . A A A A A A A A A A A A A A A A A A A A A A A A A A A A A A A A A A A A A A    
619	. . T T T T T T T T T T T T T T T T T T T T T T T T T T T T . T T T T T T T T T T T T T T T T T T T T T T T T T T T T T T T T T T T T T T    
620	. . G G G G G G G G G G G G G G G G G G G G G G G G G G G G . G G G G G G G G G G G G G G G G G G G G G G G G G G G G G G G G G G G G G G    
621	. . G G G G G G G G G G G G G G G G G G G G G G G G G G G G . G G G G G G G G G G G G G G G G G G G G G G G G G G G G G G G G G G G G G G    
622	. . T T T T T T T T T T T T T T T T T T T T T T T T T T T T . T T T T T T T T T T T T T T T T T T T T T T T T T T T T T T T T T T T T T T    
623	. . A A A A A A A A A A A A A A A A A A A A A A A A A A A A . A A A A A A A A A A A A A A A A A A A A A A A A A A A A A A A A A A A A A A    
624	. . C C C C C C C C C C C C C C C C C C C C C C C C C C C C . C C C C C C C C C C C C C C C C C C C C C C C C C C C C C C C C C C C C C C    
625	. . T T T T T T T T T T T T T T T T T T T T T T T T T T T T . T T T T T T T T T T T T T T T T T T T T T T T T T T T T T T T T T T T T T T    
626	. . G G G G G G G G G G G G G G G G G G G G G G G G G G G G . G G G G G G G G G G G G G G G G G G G G G G G G G G G G G G G G G G G G G G    
627	. . T T T T T T T T T T T T T T T T T T T T T T T T T T T T . T T T T T T T T T T T T T T T T T T T T T T T T T T T T T T T T T T T T T T    
628	. . G G G G G G G G G G G G G G G G G G G G G G G G G G G G . G G G G G G G G G G G G G G G G G G G G G G G G G G G G G G G G G G G G G G    
629	. . A A A A A A A A A A A A A A A A A A A A A A A A A A A A . A A A A A A A A A A A A A A A A A A A A A A A A A A A A A A A A A A A A A A    
630	. . A A A A A A A A A A A A A A A A A A A A A A A A A A A A . A A A A A A A A A A A A A A A A A A A A A A A A A A A A A A A A A A A A A A    
631	. . T T T T T T T T T T T T T T T T T T T T T T T T T T T T . T T T T T T T T T T T T T T T T T T T T T T T T T T T T T T T T T T T T T T    
632	. . C C C C C C C C C C C C C C C C C C C C C C C C C C C C . C C C C C C C C C C C C C C C C C C C C C C C C C C C C C C C C C C C C C C    
633	. . T T T T T T T T T T T T T T T T T T T T T T T T T T T T . T T T T T T T T T T T T T T T T T T T T T T T T T T T T T T T T T T T T T T    
634	. . G G G G G G G G G G G G G G G G G G G G G G G G G G G G . G G G G G G G G G G G G G G G G G G G G G G G G G G G G G G G G G G G G G G    
635	. . T T T T T T T T T T T T T T T T T T T T T T T T T T T T . T T T T T T T T T T T T T T T T T T T T T T T T T T T T T T T T T T T T T T    
636	. . C C C C C C C C C C C C C C C C C C C C C C C C C C C C . C C C C C C C C C C C C C C C C C C C C C C C C C C C C C C C C C C C C C C    
637	. . A A A A A A A A A A A A A A A A A A A A A A A A A A A A . A A A A A A A A A A A A A A A A A A A A A A A A A A A A A A A A A A A A A A    
638	. . G G G G G G G G G G G G G G G G G G G G G G G G G G G G . G G G G G G G G G G G G G G G G G G G G G G G G G G G G G G G G G G G G G G    
639	. . A A A A A A A A A A A A A A A A A A A A A A A A A G A A . A A A A A A A A A A A A A A A A A A A A A A A A A A A A A A A A A A A A A A    
640	. . G G G G G G G G G G G G G G G G G G G G G G G G G G G G G G G G G G G G G G G G G G G G G G G G G G G G G G G G G G G G G G G G G G G    
641	. . T T T T T T T T T T T T T T T T T T T T T T T T T T T T T T T T T T T T T T T T T T T T T T T T T T T T T T T T T T T T T T T T T T T    
642	. . . T T T T T T T T T T T T T T T T T T T T T T T T T T T T T T T T T T T T T T T T T T T T T T T T T T T T T T T T T T T T T T T T T T    
643	. . . C C C C C C C C C C C C C C C C C C C C C C C C C C C C C C C C C C C C C C C C C C C C C C C C C C C C C C C C C C C C C C C C C C    
644	. . . C C C C C C C C C C C C C C C C C C C C C C C C C C C C C C C C C C C C C C C C C C C C C C C C C C C C C C C C C C C C C C C C C C    
645	. . . A A A A A A A A A A A A A A A A A A A A A A A A A A A A A A A A A A A A A A A A A A A A A A A A A A A A A A A A A A A A A A A A A A    
646	. . . A A A A A A A A A A A A A A A A A A A A A A A A A A A A A A A A A A A A A A A A A A A A A A A A A A A A A A A A A A A A A A A A A A    
647	. . . C C C C C C C C C C C C C C C C C C C C C C C C C C C C C C C C C C C C C C C C C C C C C C C C C C C C C C C C C C C C C C C C C C    
648	. . . A A A A A A A A A A A A A A A A A A A A A A A A A A A A A A A A A A A A A A A A A A A A A A A A A A A A A A A A A A A A A A A A A A    
649	. . . C C C C C C C C C C C C C C C C C C C C C C C C C C C C C C C C C C C C C C C C C C C C C C C C C C C C C C C C C C C C C C C C C C    
650	. . . G G G G G G G G G G G G G G G G G G G G G G G G G G G G G G G G G G G G G G G G G G G G G G G G G G G G G G G G G G G G G G G G G G    
651	. . . T T T T T T T T T T T T T T T T T T T T T T T T T T T T T T T T T T T T T T T T T T T T T T T T T T T T T T T T T T T T T T T T T T    
652	. . . C C C C C C C C C C C C C C C C C C C C C C C C C C C C C C C C C C C C C C C C C C C C C C C C C C C C C C C C C C C C C C C C C C    
653	. . . A A A A A A A A A A A A A A A A A A A A A A A A A A A A A A A A A A A A A A A A A A A A A A A A A A A A A A A A A A A A A A A A A A    
654	. . . T T T T T T T T T T T T T T T T T T T T T T T T T T T T T T T T T T T T T T T T T T T T T T T T T T T T T T T T T T T T T T T T T T    
655	. . . C C C C C C C C C C C C C C C C C C C C C C C C C C C C C C C C C C C C C C C C C C C C C C C C C C C C C T C C T C C C C C C C C C    
656	. . . T T T T T T T T T T T T T T T T T T T T T T T T T T T T T T T T T T T T T T T T T T T T T T T T T T T T T T T T T T T T T T T T T T    
657	. . . C C C C C C C C C C C C C C C C C C C C C C C C C C C C C C C C C C C C C C C C C C C C C C C C C C C C C C C C C C C C C C C C C C    
658	. . . A A A A A A A A A A A A A A A A A A A A A A A A A A A A A A A A A A A A A A A A A A A A A A A A A A A A A A A A A A A A A A A A A A    
659	. . . A A A A A A A A A A A A A A A A A A A A A A A A A A A A A A A A A A A A A A A A A A A A A A A A A A A A A A A A A A A A A A A A A A    
660	. . . G G G G G G G G G G G G G G G G G G G G G G G G G G G G G G G G G G G G G G G G G G G G G G G G G G G G G G G G G G G G G G G G G G    
661	. . . . G G G G G G G G G G G G G G G G G G G G G G G G G G G G G G G G G G G G G G G G G G G G G G G G G G G G G G G G G G G G G G G G G    
662	. . . . T T T T T T T T T T T T T T T T T T T T T T T T T T T T T T T T T T T T T T T T T T T T T T T T T T T T T T T T T T T T T T T T T    
663	. . . . C C C C C C C C C C C C C C C C C C C C C C C C C C C C C C C C C C C C C C C C C C C C C C C C C C C C C C C C C C C C C C C C C    
664	. . . . A A A A A A A A A A A A A A A A A A A A A A A A A A A A A A A A A A A A A A A A A A A A A A A A A A A A A A A A A A A A A A A A A    
665	. . . . C C C C C C C C C C C C C C C C C C C C C C C C C C C C C C C C C C C C C C C C C C C C C C C C C C C C C C C C C C C C C C C C C    
666	. . . . C C C C C C C C C C C C C C C C C C C C C C C C C C C C C C C C C C C C C C C C C C C C C C C C C C C C C C C C C C C C C C C C C    
667	. . . . A A A A A A A A A A A A A A A A A A A A A A A A A A A A A A A A A A A A A A A A A A A A A A A A A A A A A A A A A A A A A A A A A    
668	. . . . G G G G G G G G G G G G G G G G G G G G G G G G G G G G G G G G G G G G G G G G G G G G G G G G G G G G G G G G G G G G G G G G G    
669	. . . . A A A A A A A A A A A A A A A A A A A A A A A A A A A A A A A A A A A A A A A A A A A A A A A A A A A A A A A A A A A A A A A A A    
670	. . . . T T T T T T T T T T T T T T T T T T T T T T T T T T T T T T T T T T T T T T T T T T T T T T T T T T T T T T T T T T T T T T T T T    
671	. . . . T T T T T T T T T T T T T T T T T T T T T T T T T T T T T T T T T T T T T T T T T T T T T T T T T T T T T T T T T T T T T T T T T    
672	. . . . T T T T T T T T T T T T T T T T T T T T T T T T T T T T T T T T T T T T T T T T T T T T T T T T T T T T T T T T T T T T T T T T T    
673	. . . . C C C T C C C C C C C C C C C C C C C C C C C C C C C C C C C C C C C C C C C C C C C C C C C C C C C C C C C C C C C C C C C C C    
674	. . . . G G G . G G G G G G G G G G G G G G G G G G G G G G G G G G G G G G G G G G G G G G G G G G G G G G G G G G G G G G G G G G G G G    
675	. . . . T T T . T T T T T T T T T T T T T T T T T T T T T T T T T T T T T T T T T T T T T T T T T T T T T T T T T T T T T T T T T T T T T    
676	. . . . C C C . C C C C C C C C C C C C C C C C C C C C C C C C C C C C C C C C C C C C C C C C C C C C C C C C C C C C C C C C C C C C C    
677	. . . . A A A . A A A A A A A A A A A A A A A A A A A A A A A A A A A A A A A A A A A A A A A A A A A A A A A A A A A A A A A A A A A A A    
678	. . . . G G G . G G G G G G G G G G G G G G G G G G G G G G G G G G G G G G G G G G G G G G G G G G G G G G G G G G G G G G G G G G G G G    
679	. . . . T T T . T T T T T T T T T T T T T T T T T T T T T T T T T T T T T T T T T T T T T T T T T T T T T T T T T T T T T T T T T T T T T    
680	. . . . T T T . T T T T T T T T T T T T T T T T T T T T T T T T T T T T T T T T T T T T T T T T T T T T T T T T T T T T T T T T T T T T T    
681	. . . . C C C . C C C C C C C C C C C C C C C C C C C C C C C C C C C C C C C C C C C C C C C C C C C C C C C C C C C C C C C C C C C C C    
682	. . . . T T T . T T T T T T T T T T T T T T T T T T T T T C T T T T T T T T T T T T T T T T T T T T T T T T T T T T T T T T T T T T T T T    
683	. . . . T T T . T T T T T T T T T T T T T T T T T T T T T T T T T T T T T T T T T T T T T T T T T T T T T T T T T T T T T T T T T T T T T    
684	. . . . T T T . T T T T T T T T T T T T T T T T T T T T T T T T T T T T T T T T T T T T T T T T T T T T T T T T T T T T T T T T T T T T T    
685	. . . . T T T . T T T T T T T T T T T T T T T T T T T T T T T T T T T T T T T T T T T T T T T T T T T T T T T T T T T T T T T T T T T T T    
686	. . . . A A A . A A A A A A A A A A A A A A A A A A A A A A A A A A A A A A A A A A A A A A A A A A A A A A A A A A A A A A A A A A A A A    
687	. . . . . A . . A A A A A A A A A A A A A A A A A A A A A A A A A A A A A A A A A A A A A A A A A A A A A A A A A A A A A A A A A A A A A    
688	. . . . . G . . G G G G G G G G G G G G G G G G G G G G G G G G G G G G G G G G G G G G G G G G G G G G G G G G G G G G G G G G G G G G G    
689	. . . . . A . . A A A A A A A A A A A A A A A A A A A A A A A A A A A A A A A A A A A A A A A A A A A A A A A A A A A A A A A A A A A A A    
690	. . . . . T . . T T T T T T T T T T T T T T T T T T T T T T T T T T T T T T T T T T T T T T T T T T T T T T T T T T T T T T T T T T T T T    
691	. . . . . T . . T T T T T T T T T T T T T T T T T T T T T T T T T T T T T T T T T T T T T T T T T T T T T T T T T T T T T T T T T T T T T    
692	. . . . . G . . G G G G G G G G G G G G G G G G G G G G G G G G G G G G G G G G G G G G G G G G G G G G G G G G G G G G G G G G G G G G G    
693	. . . . . T . . T T T T T T T T T T T T T T T T T T T T T T T T T T T T T T T T T T T T T T T T T T T T T T T T T T T T T T T T T T T T T    
694	. . . . . T . . T T T T T T T T T T T T T T T T T T T T T T T T T T T T T T T T T T T T T T T T T T T T T T T T T T T T T T T T T T T T T    
695	. . . . . C . . C C C C C C C C C C C C C C C C C C C C C C C C C C C C C C C C C C C C C C C C C C C C C C C C C C C C C C C C C C C C C    
696	. . . . . T . . T T T T T T T T T T T T T T T T T T T T T T T T T T T T T T T T T T T T T T T T T T T T T T T T T T T T T T T T T T T T T    
697	. . . . . G . . G G G G G G G G G G G G G G G G G G G G G G G G G G G G G G G G G G G G G G G G G G G G G G G G G G G G G G G G G G G G G    
698	. . . . . T . . T T T T T T T T T T T T T T T T T T T T T T T T T T T T T T T T T T T T T T T T T T T T T T T T T T T T T T T T T T T T T    
699	. . . . . G . . G G G G G G G G G G G G G G G G G G G G G G G G G G G G G G G G G G G G G G G G G G G G G G G G G G G G G G G G G G G G G    
700	. . . . . A . . A A A A A A A A A A A A A A A A A A A A A A A A A A A A A A A A A A A A A A A A A A A A A A A A A A A A A A A A A A A A A    
701	. . . . . A . . A A A A A A A A A A A A A A A A A A A A A A A A A A A A A A A A A A A A A A A A A A A A A A A A A A A A A A A A A A A A A    
702	. . . . . G . . G G G G G G G G G G G G G G G G G G G G G G G G G G G G G G G G G G G G G G G G G G G G G G G G G G G G G G G G G G G G G    
703	. . . . . T . . T T T T T T T T T T T T T T T T T T T T T T T T T T T T T T T T T T T T T T T T T T T T T T T T T T T T T T T T T T T T T    
704	. . . . . C . . C C C C C C C C C C C C C C C C C C C C C C C C C C C C C C C C C C C C C C C C C C C C C C C C C C C C C C C C C C C C C    
705	. . . . . . . . G G G G G G G G G G G G G G G G G G G G G G G G G G G G G G G G G G G G G G G G G G G G G G G G G G G G G G G G G G G G G    
706	. . . . . . . . A A A A A A A A A A A A A A A A A A A A A A A A A A A A A A A A A A A A A A A A A A A A A A A A A A A A A A A A A A A A A    
707	. . . . . . . . C C C C C C C C C C C C C C C C C C C C C C C C C C C C C C C C C C C C C C C C C C C C C C C C C C C C C C C C C C C C C    
708	. . . . . . . . A A A A A A A A A A A A A A A A A A A A A A A A A A A A A A A A A A A A A A A A A A A A A A A A A A A A A A A A A A A A A    
709	. . . . . . . . A A A A A A A A A A A A A A A A A A A A A A A A A A A A A A A A A A A A A A A A A A A A A A A A A A A A A A A A A A A A A    
710	. . . . . . . . A A A A A A A A A A A A A A A A A A A A A A A A A A A A A A A A A A A A A A A A A A A A A A A A A A A A A A A A A A A A A    
711	. . . . . . . . T T T T T T T T T T T T T T T T T T T T T T T T T T T T T T T T T T T T T T T T T T T T T T T T T T T T T T T T T T T T T    
712	. . . . . . . . A A A A A A A A A A A A A A A A A A A A A A A A A A A A A A A A A A A A A A A A A A A A A A A A A A A A A A A A A A A A A    
713	. . . . . . . . A A A A A A A A A A A A A A A A A A A A A A A A A A A A A A A A A A A A A A A A A A A A A A A A A A A A A A A A A A A A A    
714	. . . . . . . . G G G G G G G G G G G G G G G G G G G G G G G G G G G G G G G G G G G G G G G G G G G G G G G G G G G G G G G G G G G G G    
715	. . . . . . . . A A A A A A A A A A A A A A A A A A A A A A A A A A A A A A A A A A A A A A A A A A A A A A A A A A A A A A A A A A A A A    
716	. . . . . . . . T T T T T T T T T T T T T T T T T T T T T T T T T T T T T T T T T T T T T T T T T T T T T T T T T T T T T T T T T T T T T    
717	. . . . . . . . A A A A A A A A A A A A A A A A A A A A A A A A C A A A A A A A A A A A A A A A A A A A A A A A A A A A A A A A A A A A A    
718	. . . . . . . . C C C C C C C C C C C C C C C C C C C C C C C C C C C C C C C C C C C C C C C C C C C C C C C C C C C C C C C C C C C C C    
719	. . . . . . . . C C C C C C C C C C C C C C C C C C C C C C C C C C C C C C C C C C C C C C C C C C C C C C C C C C C C C C C C C C C C C    
720	. . . . . . . . A A A A A A A A A A A A A A A A A A A A A A A A A A A A A A A A A A A A A A A A A A A A A A A A A A A A A A A A A A A A A    
721	. . . . . . . . G G G G G G G G G G G G G G G G G G G G G G G G G G G G G G G G G G G G G G G G G G G G G G G G G G G G G G G G G G G G G    
722	. . . . . . . . G G G G G G G G G G G G G G G G G G G G G G G G G G G G G G G G G G G G G G G G G G G G G G G G G G G G G G G G G G G G G    
723	. . . . . . . . A A A A A A A A A A A A A A A A A A A A A A A A A A A A A A A A A A A A A A A A A A A A A A A A A A A A A A A A A A A A A    
724	. . . . . . . . T T T T T T T T T T T T T T T T T T T T T T T T T T T T T T T T T T T T T T T T T T T T T T T T T T T T T T T T T T T T T    
725	. . . . . . . . A A A A A A A A A A A A A A A A A A A A A A A A A A A A A A A A A A A A A A A A A A A A A A A A A A A A A A A A A A A A A    
726	. . . . . . . . T T T T T T T T T T T T T T T T T T T T T T T T T T T T T T T T T T T T T T T T T T T T T T T T T T T T T T T T T T T T T    
727	. . . . . . . . G G G G G G G G G G G G G G G G G G G G G G G G G G G G G G G G G G G G G G G G G G G G G G G G G G G G G G G G G G G G G    
728	. . . . . . . . G G G G G G G G G G G G G G G G G G G G G G G G G G G G G G G G G G G G G G G G G G G G G G G G G G G G G G G G G G G G G    
729	. . . . . . . . A A A A G A A A A A A A A A A A A A A A A A A G G A A A A A A A A A A A A A A A A A A A A A A A A A A A A A A A A A A A A    
730	. . . . . . . . T T T T . T T T T T T T T T T T T T T T T T T T T T T T T T T T T T T T T T T T T T T T T T T T T T T T T T T T T T T T T    
731	. . . . . . . . G G G G . G G G G G G G G G G G G G G G G G G G G G G G G G G G G G G G G G G G G G G G G G G G G G G G G G G G G G G G G    
732	. . . . . . . . C C C C . C C C C C C C C C C C C C C C C C C C C C C C C C C C C C C C C C C C C C C C C C C C C C C C C C C C C C C C C    
733	. . . . . . . . C C C C . C C C C C C C C C C C C C C C C C C C C C C C C C C C C C C C C C C C C C C C C C C C C C C C C C C C C C C C C    
734	. . . . . . . . T T T T . T T T T T T T T T T T T T T T T T T T T T T T T T T T T T T T T T T T T T T T T T T T T T T T T T T T T T T T T    
735	. . . . . . . . T T T T . T T T T T T T T T T T T T T T T T T T T T T T T T T T T T T T T T T T T T T T T T T T T T T T T T T T T T T T T    
736	. . . . . . . . G G G G . G G G G G G G G G G G G G G G G G G G G G G G G G G G G G G G G G G G G G G G G G G G G G G G G G G G G G G G G    
737	. . . . . . . . G G G G . G G G G G G G G G G G G G G G G G G G G G G G G G G G G G G G G G G G G G G G G G G G G G G G G G G G G G G G G    
738	. . . . . . . . A A . A . A A A A A A A A A A A A A A A A A A A A A A A A A A A A A A A A A A A A A A A A A A A A A A A A A A A A A A A A    
739	. . . . . . . . T T . T . T T T T T T T T T T T T T T T T T T T T T T T T T T T T T T T T T T T T T T T T T T T T T T T T T T T T T T T T    
740	. . . . . . . . T T . T . T T T T T T T T T T T T T T T T T T T T T T T T T T T T T T T T T T T T T T T T T T T T T T T T T T T T T T T T    
741	. . . . . . . . C C . C . C C C C C C C C C C C C C C C C C C C C C C C C C C C C C C C C C C C C C C C C C C C T C T T C C C C C C C C C    
742	. . . . . . . . T T . T . T T T T T T T T T T T T T T T T T T T T T T T T T T T T T T T T T T T T T T T T T T T T T T T T T T T T T T T T    
743	. . . . . . . . C C . C . C C C C C C C C C C C C C C C C C C C C C C C C C C C C C C C C C C C C C C C C C C C T C C C C C C C C C C C C    
744	. . . . . . . . T T . T . T T T T T T T T T T T T T T T T T T T T T T T T T T T T T T T T T T T T T T T T T T T T T T T T T T T T T T T T    
745	. . . . . . . . G G . G . G G G G G G G G G G G G G G G G G G G G G G G G G G G G G G G G G G G G G G G G G G G G G G G G G G G G G G G G    
746	. . . . . . . . T T . T . T T T T T T T T T T T T T T T T T T T T T T T T T T T T T T T T T T T T T T T T T T T T T T T T T T T T T T T T    
747	. . . . . . . . C C . C . C C C C C C C C C C C C C C C C C C C C C C C C C C C C C C C C C C C C C C C C C C C C C C C C C C C C C C C C    
748	. . . . . . . . C C . C . C C C C C C C C C C C C C C C C C C C C C C C C C C C C C C C C C C C C C C C C C C C C C C C C C C C C C C C C    
749	. . . . . . . . C . . C . C C C C C C C C C C C C C C C C C C C C C C C C C C C C C C C C C C C C C C C C C C C C C C C C C C C C C C C C    
750	. . . . . . . . A . . A . A A A A A A A A A A A A A A A A A A A A A A A A A A A A A A A A A A A A A A A A A A A A A A A A A A A A A A A A    
751	. . . . . . . . T . . T . T T T T T T T T T T T T T T T T T T T T T T T T T T T T T T T T T T T T T T T T T T T T T T T T T T T T T T T T    
752	. . . . . . . . T . . T . T T T T T T T T T T T T T T T T T T T T T T T T T T T T T T T T T T T T T T T T T T T T T T T T T T T T T T T T    
753	. . . . . . . . A . . A . A A A A A A A A A A A A A A A A A A A A A A A A A A A A A A A A A A A A A A A A A A A A A A A A A A A A A A A A    
754	. . . . . . . . T . . A . A A A A A A A A A A A A A A A A A A A A A A A A A A A A A A A A A A A A A A A A A A A A A A A A A A A A A A A A    
755	. . . . . . . . . . . T . T T T T T T T T T T T T T T T T T T T T T T T T T T T T T T T T T T T T T T T T T T T T T T T T T T T T T T T T    
756	. . . . . . . . . . . A . A A A A A A A A A A A A A A A A A A A A A A A A A A A A A A A A A A A A A A A A A A A A A A A A A A A A A A A A    
757	. . . . . . . . . . . C . C C C C C C C C C C C C C C C C C C C C C C C C C C C C C C C C C C C C C C C C C C C C C C C C C C C C C C C C    
758	. . . . . . . . . . . T . T T T T T T T T T T T T T T T T T T T T T T T T T T T T T T T T T T T T T T T T T T T T T T T T T T T T T T T T    
759	. . . . . . . . . . . C . C C C C C C C C C C C C C C C C C C C C C C C C C C C C C C C C C C C C C C C C C C C C C C C C C C C C C C C C    
760	. . . . . . . . . . . A . A A A A A A A A A A A A A A A A A A A A A A A A A A A A A A A A A A A A A A A A A A A A A A A A A A A T A A T A    
761	. . . . . . . . . . . G . G G G G G G G G G G G G G G G G G G G G G G G G G G G G G G G G G G G G G G G G G G G G G G G G G G G G G G G G    
762	. . . . . . . . . . . A . A A A A A A A A A A A A A A A A A A A A A A A A A A A A A A A A A A A A A A A A A A A A A A A A A A A A A A A A    
763	. . . . . . . . . . . A . A A A A A A A A A A A A A A A A A A A A A A A A A A A A A A A A A A A A A A A A A A A A A A A A A A A A A A A A    
764	. . . . . . . . . . . G . G G G G G G G G G G G G G G G G G G G G G G G G G G G G G G G G G G G G G G G G G G G G G G G G G G G G G G G G    
765	. . . . . . . . . . . A . A A A A A A A A A A A A A A A A A A A A A A A A A A A A A A A A A A A A A A A A A A A A A A A A A A A A A A A A    
766	. . . . . . . . . . . C . C C C C C C C C C C C C C C C C C C C C C C C C C C C C C C C C C C C C C C C C C C C C C C C C C C C C C C C C    
767	. . . . . . . . . . . C . C C C C C C C C C C C C C C C C C C C C C C C C C C C C C C C C C C C C C C C C C C C C C C C C C C C C C C C C    
768	. . . . . . . . . . . C . C C C C C C C C C C C C C C C C C C C C C C C C C C C C C C C C C C C C C C C C C C C C C C C C C C C C C C C C    
769	. . . . . . . . . . . A . A A A A A A A A A A A A A A A A A A A A A A A A A A A A A A A A A A A A A A A A A A A A A A A A A A A A A A A A    
770	. . . . . . . . . . . T . T T T T T T T T T T T T T T T T T T T T T T T T T T T T T T T T T T T T T T T T T T T T T T T T T T T T T T T T    
771	. . . . . . . . . . . T . T T T T T T T T T T T T T T T T T T T T T T T T T T T T T T T T T T T T T T T T T T T T T T T T T T T T T T T T    
772	. . . . . . . . . . . T . T T T T T T T T T T T T T T T T T T T T T T T T T T T T T T T T T T T T T T T T T T T T T T T T T T T T T T T T    
773	. . . . . . . . . . . T . T T T T T T T T T T T T T T T T T T T T T T T T T T T T T T T T T T T T T T T T T T T T T T T T T T T T T T T T    
774	. . . . . . . . . . . . . T T T T T T T T T T T T T T T T T T T T T T T T T T T T T T T T T T T T T T T T T T T T T T T T T T T T T T T T    
775	. . . . . . . . . . . . . T T T T T T T T T T T T T T T T T T T T T T T T T T T T T T T T T T T T T T T T T T T T T T T T T T T T T T T T    
776	. . . . . . . . . . . . . T T T T T T T T T T T T T T T T T T T T T T T T T T T T T T T T T T T T T T T T T T T T T T T T T T T T T T T T    
777	. . . . . . . . . . . . . - - - - - - - - - - - - - - T - - - - - - - - - - - - - - - - - - - - - - - - - - - - - - - - - - - - - - - - -    
778	. . . . . . . . . . . . . C C C C C C C C C C C C C C C C C C C C C C C C C C C C C C C C C C C C C C C C C C C T C T T C C C C C C C C C    
779	. . . . . . . . . . . . . T T T T T T T T T T T T T T T T T T T T T T T T T T T T T T T T T T T T T T T T T T T T T T T T T T T T T T T T    
780	. . . . . . . . . . . . . T T T T T T T T T T T T T T T T T T T T T T T T T T T T T T T T T T T T T T T T T T T T T T T T T T T T T T T T    
781	. . . . . . . . . . . . . C C C C C C C C C C C C C C C C C C C C C C C C C C C C C C C C C C C C C C C C C C C T C T T C C C C C C C C C    
782	. . . . . . . . . . . . . T T T T T T T T T T T T T T T T T T T T T T T T T T T T T T T T T T T T T T T T T T T T T T T T T T T T T T T T    
783	. . . . . . . . . . . . . T T T T T T T T T T T T T T T T T T T T T T T T T T T T T T T T T T T T T T T T T T T T T T T T T T T T T T T T    
784	. . . . . . . . . . . . . C C C C C C C C C C C C C C C C C C C C C C C C C C C C C C C C C C C C C C C C C C C C C C C C C C C C C C C C    
785	. . . . . . . . . . . . . A A A A A A A A A A A A A A A A A A A A A A A A A A A A A A A A A A A A A A A A A A A A A A A A A A A A A A A A    
786	. . . . . . . . . . . . . T T T T T T T T T T T T T T T T T T T T T T T T T T T T T T T T T T T T T T T T T T T T T T T T T T T T T T T T    
787	. . . . . . . . . . . . . T T T T T T T T T T T T T T T T T T T T T T T T T T T T T T T T T T T T T T T T T T T T T T T T T T T T T T T T    
788	. . . . . . . . . . . . . G G G G G G G G G G G G G G G G G G G G G G G G G G G G G G G G G G G G G G G G G G G G G G G G G G G G G G G G    
789	. . . . . . . . . . . . . T T T T T T G T T T T T T T T T T T T T T T T T T T T T T T T T T T T T T T T T T T T T T T T T T T T T T T T T    
790	. . . . . . . . . . . . . A A A A A A . A A A A A A A A A A A A A A A A A A A A A A A A A A A A A A A A A A A A A A A A A A A A A A A A A    
791	. . . . . . . . . . . . . A A A A A A . A A A A A A A A A A A A A A A A A A A A A A A A A A A A A A A A A A A A A A A A A A A A A A A A A    
792	. . . . . . . . . . . . . T T T T T T . T T T T T T T T T T T T T T T T T T T T T T T T T T T T T T T T T T T T T T T T T T T T T T T T T    
793	. . . . . . . . . . . . . A A A A A A . A A A A A A A A A A A A A A A A A A A A A A A A A A A A A A A A A A A A A A A A A A A A A A A A A    
794	. . . . . . . . . . . . . A A A A A A . A A A A A A A A A A A A A A A A A A A A A A A A A A A A A A A A A A A A A A A A A A A A A A A A A    
795	. . . . . . . . . . . . . T T T T T T . T T T T T T T T T T T T T T T T T T T T T T T T T T T T T T T T T T T T T T T T T T T T T T T T T    
796	. . . . . . . . . . . . . A A A A A A . A A A A A A A A A A A A A A A A A A A A A A A A A A A A A A A A A A A A A A A A A A A A A A A A A    
797	. . . . . . . . . . . . . T T T T T T . T T T T T T T T T T T T T T T T T T T T T T T T T T T T T T T T T T T T T T T T T T T T T T T T T    
798	. . . . . . . . . . . . . A A A A A T . A A A A A A A A A A A A A A A A A A A A A A A T A A A A A A A A A A A A A A A A A A A A A A A A A    
799	. . . . . . . . . . . . . T T T T T T . T T T T T T T T T T T T T T T T T T T T T T T T T T T T T T T T T T T T T T T T T T T T T T T T T    
800	. . . . . . . . . . . . . T T T T T T . T T T T T T T T T T T T T T T T T T T T T T T T T T T T T T T T T T T T T T T T T T T T T T T T T    
801	. . . . . . . . . . . . . T T T T T T . T T T T T T T T T T T T T T T T T T T T T T T T T T T T T T T T T T T T T T T T T T T T T T T T T    
802	. . . . . . . . . . . . . T T T T T - . T T T T T T T T T T T T T T T T T T T T T T T - T T T T T T T T T T T T T T T T T T T T T T T T T    
803	. . . . . . . . . . . . . T T T T T - . T T T T T T T T T T T T T T T T T T T T T T T - T T T T T T T T T T T T T T T T T T T T T T T T T    
804	. . . . . . . . . . . . . - T - - - - . - T - - - - - - - - - - - - - - - - - - - - - - - - - - - - - - - - - - - - - - - - - - - - - - -    
805	. . . . . . . . . . . . . A A A A A A . A A A A A A A A A A A A A A A A A A A A A A A A A A A A A A A A A A A A A A A A A A A A A A A A A    
806	. . . . . . . . . . . . . G G G G G G . G G G G G G G G G G G G G G G G G G G G G G G G G G G G G G G G G G G G G G G G G G G G G G G G G    
807	. . . . . . . . . . . . . A A A A A A . A A A A A A A A A A A A A A A A A A A A A A A A A A A A A A A A A A A A A A A A A A A A A A A A A    
808	. . . . . . . . . . . . . T T T T T T . T T T T T T T T T T T T T T T T T T T T T T T T T T T T T T T T T T T T T T T T T T T T T T T T T    
809	. . . . . . . . . . . . . T T T T T T . T T T T T T T T T T T T T T T T T T T T T T T T T T T T T T T T T T T T T T T T T T T T T T T T T    
810	. . . . . . . . . . . . . T T T T T T . T T T T T T T T T T T T T T T T T T T T T T T T T T T T T T T T T T T T T T T T T T T T T T T T T    
811	. . . . . . . . . . . . . T T T T T T . T T T T T T T T T T T T T T T T T T T T T T T T T T T T T T T T T T T T T T T T T T T T T T T T T    
812	. . . . . . . . . . . . . G . G G G G . G G G G G G G G G G G G G G G G G G G G G G G G G G G G G G G G G G G G G G G G G G G G G G G G G    
813	. . . . . . . . . . . . . . . C C C C . C C C C C C C C C C C C C C C C C C C C C C C C C C C C C C C C C C C C C C C C C C C C C C C C C    
814	. . . . . . . . . . . . . . . C C C C . C C C C C C C C C C C C C C C C C C C C C C C C C C C C C C C C C C C C C C C C C C C C C C C C C    
815	. . . . . . . . . . . . . . . T T T T . T T T T T T T T T T T T T T T T T T T T T T T T T T T T T T T T T T T T T T T T T T T T T T T T T    
816	. . . . . . . . . . . . . . . T T T T . T T T T T T T T T T T T T T T T T T T T T T T T T T T T T T T T T T T T T T T T T T T T T T T T T    
817	. . . . . . . . . . . . . . . A A A A . A A A A A A A A A A A A A A A A A A A A A A A A A A A A A A A A A A A A A A A A A A A A A A A A A    
818	. . . . . . . . . . . . . . . T T T T . T T T T T T T T T T T T T T T T T T T T T T T T T T T T T T T T T T T T T T T T T T T T T T T T T    
819	. . . . . . . . . . . . . . . T T T T . T T T T T T T T T T T T T T T T T T T T T T T T T T T T T T T T T T T T T T T T T T T T T T T T T    
820	. . . . . . . . . . . . . . . C C C C . C C C C C C C C C C C C C C C C C C C C C C C C C C C C C C C C C C C C C C C C C C C C C C C C C    
821	. . . . . . . . . . . . . . . T . T T . T T T T T T T T T T T T T T T T T T T T T T T T T T T T T T T T T T T T T T T T T T T T T T T T T    
822	. . . . . . . . . . . . . . . T . T T . T T T T T T T T T T T T T T T T T T T T T T T T T T T T T T T T T T T T T T T T T T T T T T T T T    
823	. . . . . . . . . . . . . . . T . T T . T T T T T T T T T T T T T T T T T T T T T T T T T T T T T T T T T T T T T T T T T T T T T T T T T    
824	. . . . . . . . . . . . . . . A . A A . . A A A A A A A A A A A A A A A A A A A A A A A A A A A A A A A A A A A A A A A A A A A A A A A A    
825	. . . . . . . . . . . . . . . . . G G . . G G G G G G G G G G G G G G G G G G G G G G G G G G G G G G G G G G G G G G G G G G G G G G G G    
826	. . . . . . . . . . . . . . . . . T T . . T T T T T T T T T T T T T T T T T T T T T T T T T T T T T T T T T T T T T T T T T T T T T T T T    
827	. . . . . . . . . . . . . . . . . C C . . C C C C C C C C C C C C C C C C C C C C C C C C C C C C C C C C C C C C C C C C C C C C C C C C    
828	. . . . . . . . . . . . . . . . . T T . . T T T T T T T T T T T T T T T T T T T T T T T T T T T T T T T T T T T T T T T T T T T T T T T T    
829	. . . . . . . . . . . . . . . . . . . . . G G G G G G G G G G G G G G G G G G G G G G G G G G G G G G G G G G G G G G G G G G G G G G G G    
830	. . . . . . . . . . . . . . . . . . . . . T T T T T T T T T T T T T T T T T T T T T T T T T T T T T T T T T T T T T T T T T T T T T T T T    
831	. . . . . . . . . . . . . . . . . . . . . A A A A A A A A A A A A A A A A A A A A A A A A A A A A A A A A A A A A A A A A A A A A A A A A    
832	. . . . . . . . . . . . . . . . . . . . . G G G G G G G G G G G G G G G G G G G G G G G G G G G G G G G G G G G G G G G G G G G G G G G G    
833	. . . . . . . . . . . . . . . . . . . . . A A A A A A A A A A A A A A A A A A A A A A A A A A A A A A A A A A A A A A A A A A A A A A A A    
834	. . . . . . . . . . . . . . . . . . . . . T T T T T T T T T T T T T T T T T T T T T T T T T T T T T T T T T T T T T T T T T T T T T T T T    
835	. . . . . . . . . . . . . . . . . . . . . T T T T T T T T T T T T T T T T T T T T T T T T T T T T T T T T T T T T T T T T T T T T T T T T    
836	. . . . . . . . . . . . . . . . . . . . . A A A A A A A A A A A A A A A A A A A A A A A A A A A A A A A A A A A A A A A A A A A A A A A A    
837	. . . . . . . . . . . . . . . . . . . . . A A A A A A A A A A A A A A A A A A A A A A A A A A A A A A A A A A A A A A A A A A A A A A A A    
838	. . . . . . . . . . . . . . . . . . . . . T T T T T T T T T T T T T T T T T T T T T T T T T T T T T T T T T T T T T T T T T T T T T T T T    
839	. . . . . . . . . . . . . . . . . . . . . A A A A A A A A A A A A A A A A A A A A A A A A A A A A A A A A A A A A A A A A A A A A A A A A    
840	. . . . . . . . . . . . . . . . . . . . . T T T T T T T T T T T T T T T T T T T T T T T T T T T T T T T T T T T T T T T T T T T T T T T T    
841	. . . . . . . . . . . . . . . . . . . . . T T T T T T T T T T T T T T T T T T T T T T T T T T T T T T T T T T T T T T T T T T T T T T T T    
842	. . . . . . . . . . . . . . . . . . . . . C C C C C C C T C C C C C C C C C C C C C C C C T C C C C C C C C C C T C T C C C C C C C C C C    
843	. . . . . . . . . . . . . . . . . . . . . T T T T T T T C T T T T T T T T T T T T T T T T T T T T T T T T T T T T T T T T T T T T T T T T    
844	. . . . . . . . . . . . . . . . . . . . . A A A A A A A . A A A A A A A A A A A A A A A A A A A A A A A A A A A A A A A A A A A A A A A A    
845	. . . . . . . . . . . . . . . . . . . . . T T T T T T T . T T T T T T T T T T T T T T T T T T T T T T T T T T T T T T T T T T T T T T T T    
846	. . . . . . . . . . . . . . . . . . . . . T T T T T T T . T T T T T T T T T T T T T T T T T T T T T T T T T T T T T T T T T T T T T T T T    
847	. . . . . . . . . . . . . . . . . . . . . A A A A A A A . A A A A A A A A A A A A A A A A A A A A A A A A A A A A A A A A A A A A A A A A    
848	. . . . . . . . . . . . . . . . . . . . . T T T T T T T . T T T T T T T T T T T T T T T T T T T T T T T T T T T T T T T T T T T T T T T T    
849	. . . . . . . . . . . . . . . . . . . . . G G G G G G G . G G G G G G G G G G G G G G G G G G G G G G G G G G G G G G G G G G G G G G G G    
850	. . . . . . . . . . . . . . . . . . . . . T T T T T T T . T T T T T T T T T T T T T T T T T T T T T T T T T T T T T T T T T T T T T T T T    
851	. . . . . . . . . . . . . . . . . . . . . . T T T T T T . T T T T T T T T T T T T T T T T T T T T T T T T T T T T T T T T T T T T T T T T    
852	. . . . . . . . . . . . . . . . . . . . . . T T T T T T . T T T T T T T T T T T T T T T T T T T T T T T T T T T T T T T T T T T T T T T T    
853	. . . . . . . . . . . . . . . . . . . . . . G . . G G G . G G G G G G G G G G G G G G G G G G G G G G G G G G G G G G G G G G G G G G G G    
854	. . . . . . . . . . . . . . . . . . . . . . A . . A A A . A A A A A A A A A A A A A A A A A A A A A A A A A A A A A A A A A A A A A A A A    
855	. . . . . . . . . . . . . . . . . . . . . . T . . T T T . T T T T T T T T T T T T T T T T T T T T T T T T T T T T T T T T T T T T T T T T    
856	. . . . . . . . . . . . . . . . . . . . . . A . . A A A . A A A A A A A A A A A A A A A A A A A A A A A A A A A A A A A A A A A A A A A A    
857	. . . . . . . . . . . . . . . . . . . . . . A . . A A A . A A A A A A A A A A A A A A A A A A A A A A A A A A A A A A A A A A A A A A A A    
858	. . . . . . . . . . . . . . . . . . . . . . T . . T T T . T T T T T T T T T T T T T T T T T T T T T T T T T T T T T T T T T T T T T T T T    
859	. . . . . . . . . . . . . . . . . . . . . . G . . G G G . G G G G G G G G G G G G G G G G G G G G G G G G G G G G G G G G G G G G G G G G    
860	. . . . . . . . . . . . . . . . . . . . . . T . . T T T . T T T T T T T T T T T T T T T T T T T T T T T T T T T T T T T T T T T T T T T T    
861	. . . . . . . . . . . . . . . . . . . . . . A . . A A A . A A A A A A A A A A A A A A A A A A A A A A A A A A A A A A A A A A A A A A A A    
862	. . . . . . . . . . . . . . . . . . . . . . T . . T T T . T T T T T T T T T T T T T T T T T T T T T T T T T T T T T T T T T T T T T T T T    
863	. . . . . . . . . . . . . . . . . . . . . . T . . T T T . T T T T T T T T T T T T T T T T T T T T T T T T T T T T T T T T T T T T T T T T    
864	. . . . . . . . . . . . . . . . . . . . . . T . . T T T . T T T T T T T T T T T T T T T T T T T T T T T T T T T T T T T T T T T T T T T T    
865	. . . . . . . . . . . . . . . . . . . . . . T . . T T T . T T T T T T T T T T T T T T T T T T T T T T T T T T T T T T T T T T T T T T T T    
866	. . . . . . . . . . . . . . . . . . . . . . T . . T T T . T T T T T T T T T T T T T T T T T T T T T T T T T T T T T T T T T T T T T T T T    
867	. . . . . . . . . . . . . . . . . . . . . . A . . T A A . A A A A A A A A A A A A A A A A A A A A A A A A A A A A A A A A A A A A A A A A    
868	. . . . . . . . . . . . . . . . . . . . . . C . . . C C . C C C C C C C C C C C C C C C C C C C C C C C C C C C C C C C C C C C C C C C C    
869	. . . . . . . . . . . . . . . . . . . . . . A . . . A A . A A A A A A A A A A A A A A A A A A A A A A A A A A A A A A A A A A A A A A A A    
870	. . . . . . . . . . . . . . . . . . . . . . T . . . T T . T T T T T T T T T T T T T T T T T T T T T T T T T T T T T T T T T T T T T T T T    
871	. . . . . . . . . . . . . . . . . . . . . . A . . . A A . A A A A A A A A A A A A A A A A A A A A A A A A A A A A A A A A A A A A A A A A    
872	. . . . . . . . . . . . . . . . . . . . . . A . . . A A . A A A A A A A A A A A A A A A A A A A A A A A A A A A A A A A A A A A A A A A A    
873	. . . . . . . . . . . . . . . . . . . . . . T . . . T T . T T T T T T T T T T T T T T T T T T T T T T T T T T T T T T T T T T T T T T T T    
874	. . . . . . . . . . . . . . . . . . . . . . G . . . G G . G G G G G G G G G G G G G G G G G G G G G G G G G G G G G G G G G G G G G G G G    
875	. . . . . . . . . . . . . . . . . . . . . . T . . . T T . T T T T T T T T T T T T T T T T T T T T T T T T T T T T T T T T T T T T T T T T    
876	. . . . . . . . . . . . . . . . . . . . . . A . . . A A . A A A A A A A A A A A A A A A A A A A A A A A A A A A A A A A A A A A A A A A A    
877	. . . . . . . . . . . . . . . . . . . . . . T . . . T T . T T T T T T T T T T T T T T T T T T T T T T T T T T T T T T T T T T T T T T T T    
878	. . . . . . . . . . . . . . . . . . . . . . A . . . A A . A A A A A A A A A A A A A A A A A A A A A A A A A A A A A A A A A A A A A A A A    
879	. . . . . . . . . . . . . . . . . . . . . . T . . . T T . T T T T T T T T T T T T T T T T T T T T T T T T T T T T T T T T T T T T T T T T    
880	. . . . . . . . . . . . . . . . . . . . . . G . . . G G . G G G G G G G G G G G G G G G G G G G G G G G G G G G G G G G G G G G G G G G G    
881	. . . . . . . . . . . . . . . . . . . . . . C . . . C C . C C C C C C C C C C C C C C C C C C C C C C C C C C C C C C C C C C C C C C C C    
882	. . . . . . . . . . . . . . . . . . . . . . A . . . A A . A A A A A A A A A A A A A A A A A A A A A A A A A A A A A A A A A A A A A A A A    
883	. . . . . . . . . . . . . . . . . . . . . . . . . . A . . A A A A A A A A A A A A A A A A A A A A A A A A A A A A A A A A A A A A A A A A    
884	. . . . . . . . . . . . . . . . . . . . . . . . . . T . . T T T T T T T T T T T T T T T T T T T T T T T T T T T T T T T T T T T T T T T T    
885	. . . . . . . . . . . . . . . . . . . . . . . . . . A . . A A A A A A A A A A A A A A A A A A A A A A A A A A A A A A A A A A A A A A A A    
886	. . . . . . . . . . . . . . . . . . . . . . . . . . A . . A A A A A A A A A A A A A A A A A A A A A A A A A A A A A A A A A A A A A A A A    
887	. . . . . . . . . . . . . . . . . . . . . . . . . . G . . G G G G G G G G G G G G G G G G G G G G G G G G G G G G G G G G G G G G G G G G    
888	. . . . . . . . . . . . . . . . . . . . . . . . . . A . . A A A A A A A A A A A A A A A A A A A A A A A A A A A A A A A A A A A A A A A A    
889	. . . . . . . . . . . . . . . . . . . . . . . . . . G . . G G G G G G G G G G G G G G G G G G G G G G G G G G G G G G G G G G G G G G G G    
890	. . . . . . . . . . . . . . . . . . . . . . . . . . C . . C C C C C C C C C C C C C C C C C C C C C C C C C C C C C C C C C C C C C C C C    
891	. . . . . . . . . . . . . . . . . . . . . . . . . . . . . C C C C C C C C C C C C C C C C C C C C C C C C C C C C C C C C C C C C C C C C    
892	. . . . . . . . . . . . . . . . . . . . . . . . . . . . . C C C C C C C C C C C C C C C C C C C C C C C C C C C C C C C C C C C C C C C C    
893	. . . . . . . . . . . . . . . . . . . . . . . . . . . . . T T T T T T T T T T T T T T T T T T T T T T T T T T T T T T T T T T T T T T T T    
894	. . . . . . . . . . . . . . . . . . . . . . . . . . . . . C C C C C C C C C C C C C C C C C C C C C C C C C C C C C C C C C C C C C C C C    
895	. . . . . . . . . . . . . . . . . . . . . . . . . . . . . T T T T T T T T T T T T T T T T T T T T T T T T T T T T T T T T T T T T T T T T    
896	. . . . . . . . . . . . . . . . . . . . . . . . . . . . . A A A A A A A A A A A A A A A A A A A A A A A A A A A A A A A A A A A A A A A A    
897	. . . . . . . . . . . . . . . . . . . . . . . . . . . . . A A A A A A A A A A A A A A A A A A A A A A A A A A A A A A A A A A A A A A A A    
898	. . . . . . . . . . . . . . . . . . . . . . . . . . . . . C C C C C C C C C C C C C C C C C C C C C C C C C C C C C C C C C C C C C C C C    
899	. . . . . . . . . . . . . . . . . . . . . . . . . . . . . T T T T T T T T T T T T T T T T T T T T T T T T T T T T T T T T T T T T T T T T    
900	. . . . . . . . . . . . . . . . . . . . . . . . . . . . . G G G G G G G G G G G G G G G G G G G G G G G G G G G G G G G G G G G G G G G G    
901	. . . . . . . . . . . . . . . . . . . . . . . . . . . . . T T T T T T T T T T T T T T T T T T T T T T T T T T T T T T T T T T T T T T T T    
902	. . . . . . . . . . . . . . . . . . . . . . . . . . . . . A A A A A A A A A A A A A A A A A A A A A A A A A A A A A A A A A A A A A A A A    
903	. . . . . . . . . . . . . . . . . . . . . . . . . . . . . T T T T T T T T T T T T T T T T T T T T T T T T T T T T T T T T T T T T T T T T    
904	. . . . . . . . . . . . . . . . . . . . . . . . . . . . . T T T T T T T T T T T T T T T T T T T T T T T T T T T T T T T T T T T T T T T T    
905	. . . . . . . . . . . . . . . . . . . . . . . . . . . . . T T T T T T T T T T T T T T T T T T T T T T T T T T T T T T T T T T T T T T T T    
906	. . . . . . . . . . . . . . . . . . . . . . . . . . . . . T T T T T T T T T T T T T T T T T T T T T T T T T T T T T T T T T T T T T T T T    
907	. . . . . . . . . . . . . . . . . . . . . . . . . . . . . T T T T T T T T T T T T T T T T T T T T T T T T T T T T T T T T T T T T T T T T    
908	. . . . . . . . . . . . . . . . . . . . . . . . . . . . . T T T T T T T T T T T T T T T T T T T T T T T T T T T T T T T T T T T T T T T T    
909	. . . . . . . . . . . . . . . . . . . . . . . . . . . . . A A A A A A A A A A A A A A A A A A A A A A A A A A A A A A A A A A A A A A A A    
910	. . . . . . . . . . . . . . . . . . . . . . . . . . . . . A A A A A A A A A A A A A A A A A A A A A A A A A A A A A A A A A A A A A A A A    
911	. . . . . . . . . . . . . . . . . . . . . . . . . . . . . A A A A A A A A A A A A A A A A A A A A A A A A A A A A A A A A A A A A A A A A    
912	. . . . . . . . . . . . . . . . . . . . . . . . . . . . . T T T T T T T T T T T T T T T T T T T T T T T T T T T T T T T T T T T T T T T T    
913	. . . . . . . . . . . . . . . . . . . . . . . . . . . . . C C C C C C C C C C C C C C C C C C C C C C C C C C C C C C T C C C C C C C C C    
914	. . . . . . . . . . . . . . . . . . . . . . . . . . . . . T T T T T T T T T T T T T T T T T T T T T T T T T T T . T T . T T T T T T T T T    
915	. . . . . . . . . . . . . . . . . . . . . . . . . . . . . A A A A A A A A A A A A A A A A A A A A A A A A A A A . A A . A A A A A A A A A    
916	. . . . . . . . . . . . . . . . . . . . . . . . . . . . . A A A A A A A A A A A A A A A A A A A A A A A A A A A . A A . A A A A A A A A A    
917	. . . . . . . . . . . . . . . . . . . . . . . . . . . . . A A A A A A A A A A A A A A A A A A A A A A A A A A A . A A . A A A A A A A A A    
918	. . . . . . . . . . . . . . . . . . . . . . . . . . . . . T T T T T T T T T T T T T T T T T T T T T T T T T T T . T T . T T T T T T T T T    
919	. . . . . . . . . . . . . . . . . . . . . . . . . . . . . A A A A A A A A A A A A A A A A A A A A A A A A A A A . A A . A A A A A A A A A    
920	. . . . . . . . . . . . . . . . . . . . . . . . . . . . . T T T T T T T T T T T T T T T T T T T T T T T T T T T . T T . T T T T T T T T T    
921	. . . . . . . . . . . . . . . . . . . . . . . . . . . . . C C C C C C C C C C C C C C C C C C C C C C C C C C C . C C . C C C C C C C C C    
922	. . . . . . . . . . . . . . . . . . . . . . . . . . . . . T T T T T T T T T T T T T T T T T T T T T T T T T T T . T T . T T T T T T T T T    
923	. . . . . . . . . . . . . . . . . . . . . . . . . . . . . A A A A A A A A A A A A A A A A A A A A A A A A A A A . A A . A A A A A A A . A    
924	. . . . . . . . . . . . . . . . . . . . . . . . . . . . . G G G G G G G G G G G G G G G G G G G G G G G G G G G . G G . G G G G G G G . G    
925	. . . . . . . . . . . . . . . . . . . . . . . . . . . . . A A A A A A A A A A A A A A A A A A A A A A A A . A A . A A . A A A A A A A . A    
926	. . . . . . . . . . . . . . . . . . . . . . . . . . . . . A A A A A A A A A A . A A A A A A A A A A A A A . A A . A A . A A A A A A A . A    
927	. . . . . . . . . . . . . . . . . . . . . . . . . . . . . T T T T T T T T T T . T T T T T T T T T T T T T . T T . T T . T T T T T T A . T    
928	. . . . . . . . . . . . . . . . . . . . . . . . . . . . . A A A A A A A A A A . A A A A A A A A A A A A A . A A . A A . A A A A A A . . A    
929	. . . . . . . . . . . . . . . . . . . . . . . . . . . . . A A A A A A A A A A . A A A A A A A A A A A A A . A A . A A . A A A A A A . . A    
930	. . . . . . . . . . . . . . . . . . . . . . . . . . . . . A A A A A A A A A A . A A A A A A A A A A A A A . A A . A A . A A A A A A . . A    
931	. . . . . . . . . . . . . . . . . . . . . . . . . . . . . A A A A A A A A A A . A A A A A A A A A A A A A . A A . A A . A A A A A A . . A    
932	. . . . . . . . . . . . . . . . . . . . . . . . . . . . . G G G G G G G G G G . G G G G G G G G G G G G G . G G . G G . G G G G G G . . G    
933	. . . . . . . . . . . . . . . . . . . . . . . . . . . . . T T T T T T T T T T . T T T T T T T T T T T T T . T T . T T . T T T T T T . . T    
934	. . . . . . . . . . . . . . . . . . . . . . . . . . . . . A A A A A A A A A A . A A A A A A A A A A A A A . A A . A A . A A A A A A . . A    
935	. . . . . . . . . . . . . . . . . . . . . . . . . . . . . A A A A A A A A A A . A A A A A A A A A A A A A . A A . A A . A A A A A A . . A    
936	. . . . . . . . . . . . . . . . . . . . . . . . . . . . . T T T T T T T T T T . T T T T T T T T T T T T T . T T . T T . T T T T T T . . T    
937	. . . . . . . . . . . . . . . . . . . . . . . . . . . . . C C C C C C C C C C . C C C C C C C G C C C C C . C C . C C . C C C C C C . . C    
938	. . . . . . . . . . . . . . . . . . . . . . . . . . . . . A A A A A A A A A A . A A A A A A A . A A A A A . A A . A A . A A A A A A . . A    
939	. . . . . . . . . . . . . . . . . . . . . . . . . . . . . A A A A A A A A A A . A A A A A A A . A A A A A . A A . A A . A A A A A A . . A    
940	. . . . . . . . . . . . . . . . . . . . . . . . . . . . . G G G G G G G G G G . G G G G G G G . G G G G G . G G . G G . G G G G G G . . G    
941	. . . . . . . . . . . . . . . . . . . . . . . . . . . . . T T T T T T T T T T . T T T T T T T . T T T T T . T T . T T . T T T T T T . . T    
942	. . . . . . . . . . . . . . . . . . . . . . . . . . . . . C C C C C C C C C C . C C C C C C C . C C C C C . C C . C C . C C C C C C . . C    
943	. . . . . . . . . . . . . . . . . . . . . . . . . . . . . T T T T T T C C T T . T T T T T T T . T T T T T . T T . T T . T C T T T T . . T    
944	. . . . . . . . . . . . . . . . . . . . . . . . . . . . . T T T T T T . . T T . T T T T T T T . T T T T T . T T . G T . T . T . T T . . T    
945	. . . . . . . . . . . . . . . . . . . . . . . . . . . . . A . . . . . . . . . . . . A . . . . . . . . . . . . . . . . . . . . . . . . . A    
946	. . . . . . . . . . . . . . . . . . . . . . . . . . . . . G . . . . . . . . . . . . G . . . . . . . . . . . . . . . . . . . . . . . . . G    
947	. . . . . . . . . . . . . . . . . . . . . . . . . . . . . T . . . . . . . . . . . . C . . . . . . . . . . . . . . . . . . . . . . . . . T    
948	. . . . . . . . . . . . . . . . . . . . . . . . . . . . . C . . . . . . . . . . . . . . . . . . . . . . . . . . . . . . . . . . . . . . C
```
